# Deep learning for 3D vascular segmentation in hierarchical phase contrast tomography: a case study on kidney

**DOI:** 10.1038/s41598-024-77582-5

**Published:** 2024-11-08

**Authors:** Ekin Yagis, Shahab Aslani, Yashvardhan Jain, Yang Zhou, Shahrokh Rahmani, Joseph Brunet, Alexandre Bellier, Christopher Werlein, Maximilian Ackermann, Danny Jonigk, Paul Tafforeau, Peter D. Lee, Claire L. Walsh

**Affiliations:** 1 Department of Mechanical Engineering, University College London, London, UK; 2 European Synchrotron Radiation Facility, Grenoble, France; 3 Department of Intelligent Systems Engineering, Luddy School of Informatics, Computing, and Engineering, Indiana University, Bloomington, USA; 4 Centre for Medical Image Computing, University College London, London, UK; 5 Laboratoire d’Anatomie Des Alpes Françaises, Grenoble, France; 6 Institute of Pathology, Hannover Medical School, Carl-Neuberg-Straße 1, 30625 Hannover, Germany; 7 Institute of Functional and Clinical Anatomy, University Medical Center of the Johannes Gutenberg-University Mainz, Mainz, Germany; 8 Institute of Pathology, RWTH Aachen University, Pauwelsstrasse 30, 52074 Aachen, Germany; 9 National Heart and Lung Institute, Faculty of Medicine, Imperial College London, London, UK

**Keywords:** Deep learning, X-ray tomography, Semantic segmentation, 3D vascular segmentation, Computer science, Kidney

## Abstract

Automated blood vessel segmentation is critical for biomedical image analysis, as vessel morphology changes are associated with numerous pathologies. Still, precise segmentation is difficult due to the complexity of vascular structures, anatomical variations across patients, the scarcity of annotated public datasets, and the quality of images. Our goal is to provide a foundation on the topic and identify a robust baseline model for application to vascular segmentation using a new imaging modality, Hierarchical Phase-Contrast Tomography (HiP-CT). We begin with an extensive review of current machine-learning approaches for vascular segmentation across various organs. Our work introduces a meticulously curated training dataset, verified by double annotators, consisting of vascular data from three kidneys imaged using HiP-CT as part of the Human Organ Atlas Project. HiP-CT pioneered at the European Synchrotron Radiation Facility in 2020, revolutionizes 3D organ imaging by offering a resolution of around 20 μm/voxel and enabling highly detailed localised zooms up to 1–2 μm/voxel without physical sectioning. We leverage the nnU-Net framework to evaluate model performance on this high-resolution dataset, using both known and novel samples, and implementing metrics tailored for vascular structures. Our comprehensive review and empirical analysis on HiP-CT data sets a new standard for evaluating machine learning models in high-resolution organ imaging. Our three experiments yielded Dice similarity coefficient (DSC) scores of 0.9523, 0.9410, and 0.8585, respectively. Nevertheless, DSC primarily assesses voxel-to-voxel concordance, overlooking several crucial characteristics of the vessels and should not be the sole metric for deciding the performance of vascular segmentation. Our results show that while segmentations yielded reasonably high scores-such as centerline DSC ranging from 0.82 to 0.88, certain errors persisted. Specifically, large vessels that collapsed due to the lack of hydrostatic pressure (HiP-CT is an ex vivo technique) were segmented poorly. Moreover, decreased connectivity in finer vessels and higher segmentation errors at vessel boundaries were observed. Such errors, particularly in significant vessels, obstruct the understanding of the structures by interrupting vascular tree connectivity. Our study establishes the benchmark across various evaluation metrics, for vascular segmentation of HiP-CT imaging data, an imaging technology that has the potential to substantively shift our understanding of human vascular networks.

## Introduction

The vascular system consists of the network of blood vessels responsible for circulating blood, delivering oxygen, and facilitating the removal of waste from tissues and organs^1^. Abnormalities in the structure or function of vascular networks can lead to, or be indicative of, various pathologies ranging from tumor growth and metastasis to strokes and cardiovascular disorders. Hence, accurate segmentation of vasculature and quantitative evaluation of its morphology are widely used to better understand these pathophysiological processes^2–5^.

Annotation of images by experts has been regarded as the segmentation gold standard, but it is time-consuming and requires specialised knowledge^6^. The success of convolutional neural networks (CNN) in classification leads researchers to employ deep learning technologies for image segmentation^7,8^. Consequently, a growing number of automated and semi-automatic vessel segmentation methods have been developed over the last years and applied in the diagnosis of diseases associated with the vascular system^4,6,9^.

However, the complexity of vascular imaging data and the anatomical variability among subjects make vessel segmentation a challenging task. Despite the clinical need, a fully automated segmentation method for 3D vascular segmentation and subsequent feature extraction has not yet been developed due to several specific challenges^10^. The main hurdles are the variations across scales, differences in individual anatomies, complexities of slender structures, a limited volume ratio (i.e. vessel/background), as well as a scarcity of publicly available labeled data. In terms of individual anatomical variations, there are significant disparities in vessel length, diameter, and tortuosity, complicating the tasks of vascular tracking and segmentation^10–13^. Additionally, narrower vessels with diameters at the border of the imaging resolution are particularly difficult to capture^10,14^.

For segmentation algorithms to have application in biomedical imaging, they must handle a broad spectrum of anatomical and sample variations. They should also be versatile enough to operate across various imaging modalities that cover different length scales and be applicable to both in vivo and ex vivo imaging. For in vivo vascular imaging, magnetic resonance angiography (MRA) is one of the most common choices due to its noninvasive nature, the absence of ionizing radiation exposure, its capability for non-contrast examination, and its ability to provide volumetric representations that highlight vascular disease^15^.

Hierarchical Phase-Contrast Tomography (HiP-CT)^16^ is an ex vivo imaging technology developed in 2020 on the BM05 beamline at the European Synchrotron Radiation Facility (ESRF)’s Extremely Brilliant Source (EBS). The technique is a propagation-based phase-contrast tomography technique that utilises the increased brilliance and resulting high spatial coherence of the European Synchrotron Radiation Facility’s upgrade to a 4th Generation X-ray source (The Extremely Brilliant Source (EBS). With HiP-CT, it is possible to resolve very small refractive index difference (density difference) in soft tissue structures, even in very large samples such as whole adult human organs. The propagation through free space of the X-rays, after interaction with the sample allow the refractive index shifts to be converted to intensity variation through single-distance phase retrieval at reconstruction^17^. HiP-CT has been used to image ex vivo whole human organs, down to resolution of some single-cell types in regions of interest (e.g. Purkinje cells in cerebellar cortex)^16^.

**Fig. 1:**
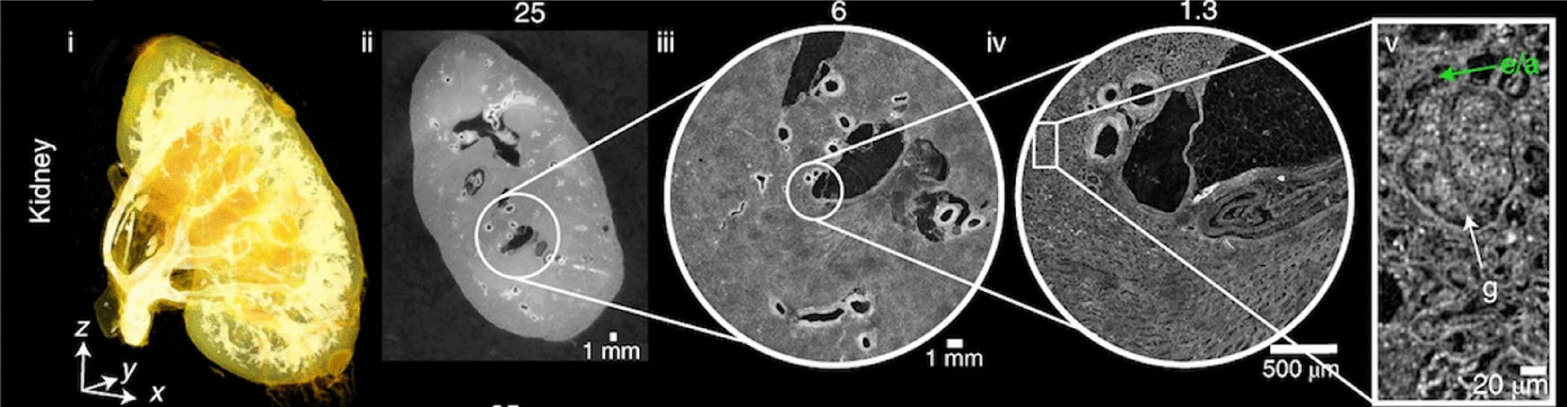
Reprint from Walsh et al.^16^, HiP-CT of kidney; a 3D rendering (i) of the whole organ is shown using scans at 25 μm per voxel. Subsequent 2D slices (ii–iv) show positions of the higher-resolution VOI relative to the previous scan. (v), Digital magnification of the highest-resolution image with annotations depicting characteristic structural features in the kidney (e/a, efferent or afferent arteriole; g, glomerulus) and in the spleen (rp, red pulp; wp, white pulp; a, arteriole; ss, splenic sinus). All images are shown using 2$\times$ binning.

A specific novelty of HiP-CT imaging as it relates to the vasculature, lies in its hierarchical nature. Whole human organs can be imaged with overview resolution of ~ 20 μm/voxel, then followed by high resolution (down to ~ 1–2 μm/voxel) regions of interest anywhere within the sample without sectioning (Fig. 1). This unique feature allows researchers to image the hierarchical structure of the vascular tree from the largest vessels down to near the capillary bed in intact human organs. This novel capability has already allowed unique insights into vascular abnormalities in COVID-19 lung lobes to be identified^18–20^, as well as vascular quantification across the whole organ^21^. Despite its advantages and suitability for vessel imaging, HiP-CT also poses many challenges for CNN-based methods of vascular segmentation:

- Large data volume (routinely $\ge$ 1Tb) require cutting-edge hardware
- Multi-scale vessels to segment
- Lack of available ground truth training data due to the novelty of the method
- Phase contrast generates fringes in intensity at vessel boundaries
- Large vessels can collapse
- No targeted vascular staining to enhance contrast between vessel lumen and surrounding tissue
- Continually developing technique with ongoing changes in image quality (e.g. improvement to resolution, SNR and CNR) result in heterogeneous datasets

In light of the opportunity and challenges that HiP-CT poses for vascular research, we review the literature on blood vessel segmentation with a particular focus on identifying current models that are most suited to the segmentation of blood vessels from HiP-CT imaging. We review segmentation on an organ-by-organ basis as the vascular and parenchymal tissue structures surrounding the vasculature differ vastly between different tissues. Our ultimate aim is to identify and then develop a model and training strategy which will provide a robust and generalisable method that can be easily adapted to segment multi-scale vascular structures across all organs imaged by HiP-CT.

The main contributions of the article are as follows:

- A comprehensive review of deep learning methods for vascular segmentation
- Creation of a Hierarchical Phase-Contrast Tomography (HiP-CT) labeled vascular dataset in the human kidney
- Implementation of the nnU-Net model, tailored for kidney vessel segmentation, using our novel Hierarchical Phase-Contrast Tomography (HiP-CT) dataset
- In-depth discussion on the challenges of blood vessel segmentation and the significance of performance metrics
- Establishment of a benchmark for future model evaluations in the context of phase contrast tomography imaging data

This paper is arranged as follows: In “Vessel segmentation” Section, deep learning models used for vessel segmentation, the datasets used in their training, and the evaluation metrics in the literature are briefly explained. In “nnU-Net for kidney vessel segmentation” Section, the most prominent method in the literature is tested on HiP-CT kidney vascular data to establish a baseline of performance and highlight specific challenges in the adaptation of these models to HiP-CT data. The findings from our experiments are presented in “Results” Section, followed by a discussion of these results in “Discussion” Section. Finally, We conclude the paper in “Conclusion and future work” Section with a summary of our conclusions and suggestions for future research directions.

## Vessel segmentation

### Modalities for 3D vascular imaging

Whilst the ideal vessel segmentation algorithm or model is robust across a range of imaging modalities, in reality, the imaging modality will impose contrast and resolution limits that must be considered in segmentation approaches as well as imaging-specific artefacts (e.g anisotropic voxels). Here, we review some of the most common imaging modalites for 3D vascular networks spanning both in vivo whole human organ imaging as well as ex vivo human tissue imaging; we contextualise the development of HiP-CT within this field.

The most widely adopted procedure for visualising vessels in human organs in vivo is angiography, i.e. where a contrast agent is injected into the vessels. The major angiographic modalities utilised in clinical practice are computed tomography angiography (CTA), magnetic resonance angiography (MRA), and digital subtraction angiography (DSA) also known as a conventional angiogram.

MRA emerged as the predominant modality in vessel segmentation studies we reviewed, as evidenced in Tables 1, 2, 3 and 4. Of the 31 deep learning-based vessel segmentation studies we reviewed, 9 employed MRA, which was primarily used for cerebral vascular evaluations due to its superior tissue contrast compared to CTA. Contrast agents used in MR and CT imaging do not cross the blood-brain barrier, making MRA the preferred choice in these cases. Commonly available datasets for these studies, such as PEGASUS^22^, MIDAS^23^, SCAPIS^24^, and 1000PLUS^25^, also utilise MRA. Additionally, 9 studies utilised CTA, which was applied to different regions including the brain, kidney, coronary, and pulmonary vessels.

MRA can be divided into three categories depending on the technique behind the image acquisition/generation: TOF (time-of-flight), PC (phase contrast), and CE (contrast-enhanced). Among those, TOF-MRA is the most frequently used non-contrast bright-blood technique for imaging the human vascular system^26,27^. As suggested by its name, TOF MRA relies on a principle known as flow-related enhancement, which happens when completely magnetised blood flows into a slab of magnetically saturated tissue whose signal has been muted by repeated RF-pulses^28^. However, the long acquisition time and high operational costs make it difficult to employ for cerebral artery visualisation^29,30^. Furthermore, MRA has been shown to overestimate stenosis compared to other modalities^31^.

CTA, on the other hand, is faster, takes a few minutes to complete, and is more accurate than MRA. However, unlike MRA, all CTAs need the administration of an IV contrast agent and involve radiation exposure, making it less safe.

DSA has been utilised as the gold standard for imaging vessels; nonetheless, this invasive and labor-intensive method is rather costly and comes with discomforts and possible dangers^32,33^. For the in vivo methods described above the image resolution is on the order of 0.5–2 mm^27,32^, with the state-of-the-art TOF techniques improving the resolution to at most 0.3–0.5 mm. Capturing the microvasculature (1–100 μm ) is only possible in vivo at very shallow depths using e.g. contrasted ultrasound^34^. Otherwise, microvascular imaging of human tissue is restricted to ex vivo techniques including optical, MRI, X-ray, or ultrasound modalites. These techniques are generally applied to smaller tissue sections^35,36^, with some notable exceptions^16,37,38^. Ex vivo techniques, capable of imaging a whole human organ down to 100 μm or less include only three examples to the authors’ knowledge: Light-sheet^37^, 7T MRI^38^ and HiP-CT^16^. Lightsheet imaging, where a fluorescence excitation beam is focused to a sheet which passes though the sample with the detector of the fluorescence emission arranged orthogonal to the illumination, relies on tissue clearing and staining to render the whole organ optically transparent and to target specific structure. High field MRI can provide higher spatial resolution but not below 100 μm. Of the three techniques, HiP-CT can perform multi-scale imaging far more simply than light sheet imaging as HiP-CT does not rely on staining of the vasculature, or clearing of the sample shortening sample preparation,^21,37^. In additon the imaging itself of a whole organ at 20 μm/voxel can be performed in as little as 1 hr compared to the MRI aquisitions 100 μm which took hundreds of hours^21,38^.

Before the arrival of complex machine learning methods, vessel segmentation relied heavily on traditional techniques such as kernel-based methods, tracking methods, mathematical morphology-based methods, and model-based methods^39^. In kernel-based methods, edge detection techniques, like the Canny or Sobel operators, identify vessel boundaries using gradient information^40^. More sophisticated approaches, such as Hessian-based methods can be categorised under model-based methods^41^. These methods utilise second-order intensity derivatives to capture vessel-like structures, and level-set methods evolve contours within the image domain to detect vessels. Active contours, or snakes, are tracking methods dynamically adjusted to capture vessel boundaries, and mathematical tools like B-splines offer precise modeling of vessel structures^42,43^. These traditional methods provided a foundation that paved the way for machine learning in the realm of medical image analysis.

With the rapid advancement of computational capabilities, a paradigm shift has taken place in the realm of image processing and machine learning: the rise of deep neural networks (NNs). These NNs, employing multi-layered architectures, extracted patterns within data through multiple levels of abstraction, and transformed the way images are understood and processed.

Among various NN architectures, CNNs have stood out as highly effective for image analysis tasks. They possess the capability to learn image features hierarchically, progressing from simple to complex, thereby eliminating the need for manual feature extraction. This inherent feature has made CNNs especially popular in the field of medical image analysis.

Recently, the landscape of computer vision has seen another transformative shift with the emergence of Transformers^44^. Initially successful in natural language tasks, Transformers have begun to find application in various computer vision challenges, prompting researchers to reevaluate the dominant role of CNNs. These advancements in computer vision have also generated significant interest in the medical imaging domain^45^. Transformers, with their ability to capture a more extensive global context compared to CNNs have now become a subject of considerable attention in the medical imaging community^46,47^.

### Deep learning models for vessel segmentation

**Table 1:** Review of the papers that applied deep learning for brain vessel segmentation.

References	Modality	Data source	No. of subj.	ML model	Input	DSC	Add’l perf. metrics
Livne et al.^48^	TOF MRA	Private (PEGASUS)	66	U-Net	2D patches	0.88	95HD = $\sim$ 47 voxels an AVD = $\sim$ 0.4 voxels
Phellan et al.^49^	TOF MRA	Private	5	2D CNN	2D patches	Between 0.764 0.786	–
Hilbert et al.^50^	TOF MRA	Private (PEGASUS+ 7UP+ 1000Plus)	264	3D CNN (BRAVE-NET)	3D patches	0.931	95HD = 29.153, AVD = 0.165
Patel et al.^51^	DSA	Private	100	3D CNN (DeepMedic) 3D U-Net	3D patches	0.94 ± 0.02 0.92 ± 0.02 respectively	CAL = 0.84±0.07 0.79 ± 0.06 respectively
Tetteh et al.^52^	TOF MRA $\mu$CTA	Publicly available	Synthetic data	NN with 2D orthogonal cross-hair filters (DeepVesselNet)	3D volumes	0.79	Prec = 0.77 Recall = 0.82
Garcia et al.^53^	3DRA	Private	5	3DUNet-based architectures	3D patches	0.80 ± 0.06	Prec = 0.75 ± 0.10 and Recall = 0.90 ± 0.07
Vos et al.^54^	TOF-MRA	Private	69	2D and 3D U-Net	2D and 3D patches	0.74 ± 0.17 0.72 ± 0.15 respectively	MHD = 47.6 ± 40.4 5 81.3 ± 57.0 respectively
Chatterjee et al.^55^	TOF MRI	Not explicity stated	11	U-Net MSS	3D patches	0.79 ± 0.091	IOU = 65.89 ± 1.25
Zhang and Chen^56^	TOF-MRA	Not explicity stated	42	DD-Net	3D patches	0.67	Sensitivity = 67.86 IOU = 33.66
B.Zhang et al.^57^	TOF-MRA	Publicly available (MIDAS)	109	DD-CNN	3D patches	0.93	PPV = 96.4730, Sensitivity = 90.1443, Acc = 99.9463
Lee et al.^58^	MRA	Private (SNUBH)	26	2D U-Net with LSTM (Spider U-Net)	‘2D images	0.793	IOU = 74.3
Quintana et al.^59^	TOF-MRA	Private (UNAM)	4	Dual U-Net-Based cGAN	2D images	0.872	Prec = 0.895

*CNN* convolutional neural network, *GAN* generative adversarial network, *cGAN* conditional GAN, *DD-Net* dense-dilated neural network, *LSTM* long short-term memory, *U-Net MSS* multi-scale supervised U-Net, *TOF MRA* time of flight magnetic resonance angiography, *DSA* digital subtraction angiography, $\mu$*CTA* micro-computed tomography angiography, *3DRA* 3D rotational angiographies, *DSC* Dice similarity coefficient, *HD* Hausdorff distance, *AVD* average distance, *Prec* Precision, *MHD* Mahalanobis distance, *IOU* intersection over union, *PPV* positive predictive value, *CAL* connectivity-area-length.

**Table 2:** Review of the papers that applied deep learning for kidney vessel segmentation.

References	Modality	Data source	No. of subj.	ML model	Input	DSC	Add’l perf. metrics
Karpinski et al.^60^	WSI	Publicly available	35	2D UNet with Resnet34 backbone	2D images	–	Acc: 0.893
He et al.^61^	CT	Private	170	DPA-DenseBiasNet	3D volumes	0.861	MCD:1.976
Taha et al.^62^	CT	Private	99	Kid-Net (a 3D CNN)	3D patches	–	F1 score: 0.72 (artery); 0.67 (vein)
He at al.^63^	CTA	Private	122	EnMcGAN	3D patches	0.89 ± 0.6 (artery); 0.77 ± 0.12 (vein)	–
Zhang et al.^64^	CT	Publicly available	392	DPA-DenseBiasNet	2D images	0.884	–
Xu et al.^65^	Micro-CT	Private	8	CycleGAN	3D patches	0.768 ± 0.3	Acc: 0.992
Li et al.^66^	CT	Publicly available	35	DUP-Net	3D patches	0.883	Precision:0.911; Recall:0.858

*CNN* convolutional neural network, *GAN* generative adversarial network, *WSI* whole slide imaging, *DPA* deep priori anatomy, *MCD* mean centerline distance, *EnMcGAN* ensemble multi-condition GAN, *DUP-Net* double UPoolFormer networks, *DSC* Dice similarity coefficient.

**Table 3:** Review of the papers that applied deep learning for coronary vessel segmentation.

References	Modality	Data source	No. of subj.	ML model	Input	DSC	Add’l perf. metrics
Dong et al.^67^	CCTA	Private	338	Di-Vnet	3D patches	0.902	Prec = 0.921, Recal l= 0.97
Gao et al.^68^	XCA	Private	130	GBDT	2D images	–	F1 = 0.874, Sen = 0.902, Spec = 0.992
Wolterink et al.^69^	CCTA	Publicly available	18	GCN	2D images	0.74	MSD = 0.25 mm
Li et al.^70^	CCTA	Private	243	2D U-Net with 3DNet	2D images	0.771 ± 0.021	AUC = 0.737
Song et al.^71^	CCTA	Private	68	3D FFR U-Net	3D patches	0.816	Prec = 0.77, Recall = 0.87
Zeng et al.^72^	CCTA	Publicly available	1000	3D-UNet	3D volumes	0.82	–

*DSC* Dice similarity coefficient, *Prec* Precision, *Sen* sensitivity, *Spec* specificity, *GBDT* gradient boosting decision tree, *CCTA* coronary computed tomographic angiography, *XCA* X-ray coronary angiography, *GCN* graph convolutional networks, *MSD* mean surface distance, *AUC* area under the receiver operating characteristic curve, *3D FFR U-Net* 3D feature fusion and rectification U-Net.

**Table 4:** Review of the papers that applied deep learning for pulmonary vessel segmentation.

References	Modality	Data source	No. of subj.	ML model	Input	DSC	Add’l perf. metrics
Tan et al.^73^	CT and CTA	Publicly available	16	2D-3D U-Net nnU-net	2D images 3D volume	0.786, 0.797, 0.77 (on CT)	OR = 0.281, 0.285, 0.304
Nam et al.^74^	CTA	Private	104	3D U-Net	3D patches	0.915 ± 0.31	AUC = 0.995
Guo et al.^75^	CT	Private	50	3D CNN	3D patches	0.943	–
Xu et al.^76^	CT	Private		2D CNN		–	–
Nardelli et al.^77^	CT	Publicly available	55	3D CNN	2D and 3D patches	–	Sen = 0.93 Prec = 0.83
Wu et al.^78^	CT	Private	143	MSI-U-Net	3D volume	0.7168	Sen = 0.7234, Prec = 0.7893

*CNN* convolutional neural network, *CT* computed tomography, *CTA* computed tomography angiography, *MSI-U-Net* multi-scale interactive U-Net, *OR* over segmentation rate, *AUC* area under the receiver operating characteristic curve, *Sen* sensitivity, *Spec* specificity, *Prec* precision.

Whilst the acquisition of these large vascular network datasets is becoming increasingly fast, automated segmentation has also been a rapidly developing area of research^79^. Before the introduction of machine learning models, traditional vessel segmentation methods such as the Frangi^80^ and Sato^81^ filters were widely employed for their ability to enhance tubular structures in medical images. These filters have played a crucial role in extracting vascular structures, particularly in medical imaging contexts like angiography, and continue to be referenced as foundational techniques in vessel segmentation. These techniques fundamentally rely on the gradient of intensity along the long axis of the vessel being smaller than the intensity gradient orthogonal to the vessel where the vessel wall is crossed. Due to the label-free nature of HiP-CT and to the effects of partial blood infilling, large vessel collapse, and intensity gradients that can occur in tomographic reconstruction such as ring artefact removal^82^, such gradient-based approaches were unsuccessful when applied in an early investigation into HiP-CT vessel segmentation.

In this section, we delve into the latest deep-learning (DL) strategies used for blood vessel segmentation. We’ve grouped the DL-based techniques for segmenting vessels under three primary network structures: Convolutional Neural Networks, Generative Adversarial Networks, and Vision Transformers.

#### Convolutional neural network based models (CNNs)

Traditional CNNs have been foundational in vessel segmentation before the widespread adoption of architectures like U-Net. These neural networks, based on fundamental concepts of learning features and building hierarchical representations, leverage the presence of spatial structures and patterns inherent in image data. Unlike the Fully Convolutional Networks (FCN) that perform dense predictions, traditional CNNs often operate on patches or regions, aiming at classifying central pixels or aggregates.

Yao et al. used a 2D CNN architecture to extract blood vessels from fundus images^83^. The output of their CNN architecture is the confidence level of each pixel being blood vessels. In 2018, Tetteh et al. developed an architecture called DeepVesselNet. Their design leverages 2-D orthogonal cross-hair filters using 3-D context information while minimizing computational demands. Addinionally, they introduce a class balancing cross-entropy loss function with false positive rate correction specifically tailored to address the prevalent issues of significant class imbalances and high false positive rates observed with traditional loss functions^52^.

Cervantes-Sanchez et al. trained a multilayer perceptron with X-ray Coronary Angiography (XCA) images enhanced by using Gaussian filters in the spatial domain and Gabor filters in the frequency domain for segmentation of coronary arteries in X-ray angiograms^84^. Nasr-Esfahani et al. presented a multi-stage model where a patch around each pixel is fed into a trained CNN to determine whether the pixel is of vessel or background regions^85^. Samuel and Veeramalai proposed a two-stage vessel extraction framework to learn well-defined vessel features from global features (learned by the pre-trained VGG-16 base network) using the Vessel Specific Convolutional (VSC) blocks, Skip chain Convolutional (SC) layers, and feature map summations^86^. In 2021, Iyer et al. developed a new CNN for angiographic segmentation: AngioNet, which combines an Angiographic Processing Network (APN) with Deeplabv3+ because of its ability to approximate more complex functions. The APN was tailored to tackle several challenges specific to angiographic segmentation, including low-contrast images and overlapping bony structures^87^.

**Fig. 2:**
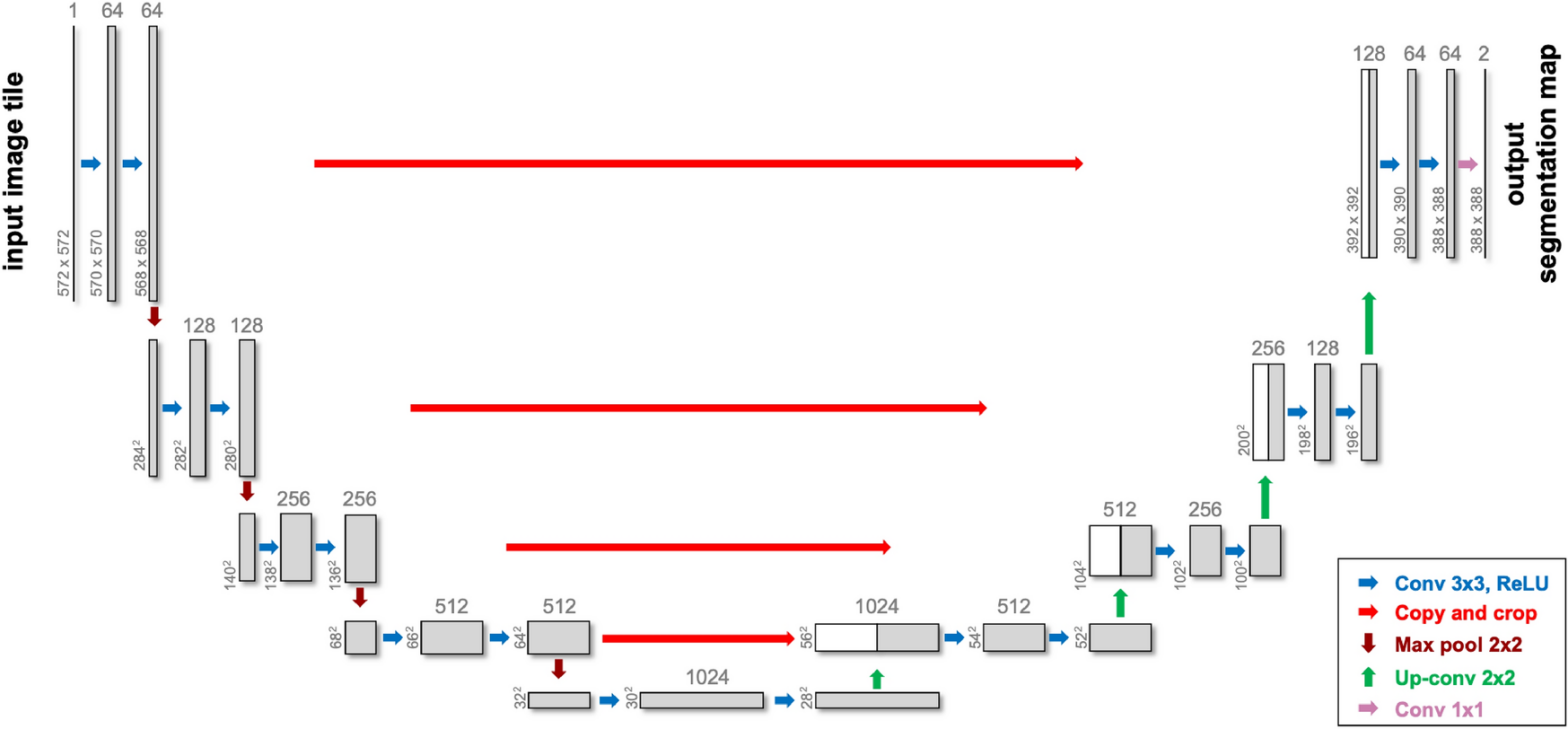
A sample U-Net architecture used in medical image segmentation tasks-modified after^88^.

*Fully convolutional neural network (FCN) based models: U-Net and its variants*

Fully Convolutional Networks (FCNs) are a subtype of CNNs specialised in semantic segmentation tasks. Deep learning techniques have revolutionised medical image segmentation, achieving precise pixel-level classification with the help of FCN^89^. FCNs are designed to produce an output of the same size as the input (i.e., a pixel-wise map). They replace the dense layers with convolutional layers. Expanding upon FCN’s foundation, many researchers have pioneered advanced 2D fully convolutional neural networks, including U-Net, SegNet, DeepLab, and PSPNet^88,90–92^. Khan et al. presented a fully convolutional network called RC-Net for retinal image segmentation. The network itself is relatively small and the number of filters per layer is optimised to reduce feature overlapping and complexity as compared to alternatives elsewhere in the literature. In the model, they kept pooling operations to a minimum and integrated skip connections into the network to preserve spatial information^93^.

U-Net, distinguished by its unique U-shaped architecture, has become particularly popular for medical image segmentation tasks, especially when the dataset is limited^88^. It leverages skip-layer connections combined with encoder-decoder multi-scale features, enhancing segmentation precision. An example of a Unet architecture is illustrated in Fig. 2.

Given U-Net’s success in medical image segmentation, Livne et al. streamlined it by halving the channels in each layer, coining it “half U-Net” for brain vessel segmentation^48^. Even though there has been extensive exploration of 2D methodologies in the literature (see Tables 1, 2, 3 and 4), especially in coronary vessel segmentation (see Table 3), it is observed that relying solely on 2D segmentation networks may overlook inter-slice relationships, leading to subpar segmentation^78^. Additionally, given the nonplanar nature of blood vessels, attempting to segment them without adequate volumetric information, such as on a single slice or in 2.5D scenarios, poses challenges. Although direct comparison of different studies is hampered due to the use of different datasets, the results from the studies detailed in see Tables 1, 2, 3 and 4 suggest that 3D models outperform 2D models. Specifically, 3D models achieve an average Dice score coefficient (DSC) of 0.83, compared to the 0.79 average for 2D models, underscoring the significance of interslice relationships.

Consequentially, researchers have proposed 3D segmentation networks like 3D U-Net, V-Net, and VoxResNet^94–96^. Huang et al. utilised 3D U-Net with data augmentation for liver vessel segmentation and introduced a weighted DSC loss function to address the voxel imbalance between vessels and other tissues^97^.

To enhance the segmentation abilities of the U-Net network, several modifications have been proposed. One notable advancement is the attention U-Net, introduced by Oktay et al., which is designed to suppress irrelevant regions in an input image while highlighting salient features useful for a specific task^98^. Bahdanau et al. originally conceived the attention mechanism to tackle the challenges arising from using a fixed-length encoding vector, which restricted the decoder’s access to input information^99^. This mechanism mimics human attention, enabling the model to concentrate on specific input areas while generating an output. The core idea behind this mechanism is to assign different weights to different parts of the input data, indicating how much “attention” each part should receive relative to others when producing a specific output.

Building upon the U-Net framework, Zhou and colleagues implemented U-Net ++ with the premise that the network would face a more straightforward learning challenge when the feature maps from both the decoder and encoder networks have semantic similarities^100^. With this goal in mind, they reconfigured the skip pathways to diminish the semantic disparity between the encoder’s and decoder’s feature maps.

Sanches et al. melded 3D U-Net and Inception, dubbing it “Uception” for brain vessel segmentation^101^. Dong and colleagues introduced a cascaded residual attention U-Net, termed CRAUNet, for a layered analysis of retinal vessel segmentation. The architecture capitalizes on the advantages of U-Net, coupled with cascaded atrous convolutions and residual blocks that are further enhanced by squeeze-and-excitation features^102^. Drawing inspiration from U-Net and DropBlock, Guo et al. introduced the Structured Dropout U-net (SD-Unet) for coronary vessel segmentation. This design amalgamates the U-Net and DropBlock frameworks to omit specific semantic details, thereby preventing the network from overfitting^103^.In 2021, Pan et al. adopted a 3D Dense-U-Net model and replaced the standard DSC loss function with the focal loss function to tackle the issue of class imbalance to achieve fully automated segmentation of the coronary artery^104^. In 2022, Wu and colleagues introduced the Multi-Scale Interactive U-Net (MSI-U-Net), an enhancement of the 3D U-Net that enhances the precision of segmenting smaller vessels^78^. They offered a strategy for interacting with information at multiple scales, where features are transferred among vessels of varying sizes through shared convolution kernel parameters, strengthening the relationship between small, medium, and large vessels in lung CT scans.

Moreover, to address the challenge of extensive annotation requirements in vessel segmentation, Koziński et al.^105^ proposed a method that utilizes 2D tracing to reduce the annotation effort for 3D deep delineation of linear structures. Similarly, Dang et al.^106^ introduced Vessel-CAPTCHA, an efficient learning framework designed to streamline vessel annotation and segmentation tasks.

nnU-Net^107^ is one of the most widely utilised frameworks for vessel segmentation tasks, consistently delivering impressive results. It is considered the state-of-the-art for a wide variety of medical image segmentation tasks, with the original or variant of nnU-Net winning international biomedical challenges across 11 tasks^107,108^. It has also recently been proposed as the network for the standardised evaluation of continual segmentation^108^. Rather than introducing a novel model architecture, nnU-Net is designed to autonomously adapt and configure the entire segmentation framework, encompassing preprocessing, network architecture, training, and post-processing, to suit any new task. This deep learning framework is known for its flexibility, scalability, and ease of use in tackling various medical imaging segmentation problems. To this end, it is frequently used to benchmark new model approaches, or combined with other models as in ensembles to improve performance.

It should be noted that as Moccia et al.^4^ and Garcia et al.^53^ recently mentioned, the success of the segmentation approaches is highly influenced not only by the algorithm but also by factors such as imaging modalities the presence/absence of noise or artifacts, and the anatomical region of interest. This makes direct comparisons among the studies in the literature challenging. Although different modalities, such as CTA and MRA, were used, the majority of the reviewed papers (71%) relied on private data, making it difficult to replicate or compare their findings.

The data presented in see Tables 1, 2, 3 and 4 reveals a predominant use of U-Net or variants of CNNs in the majority of studies, accounting for 80% of the cases.

#### Generative adversarial networks (GANs)

**Fig. 3:**
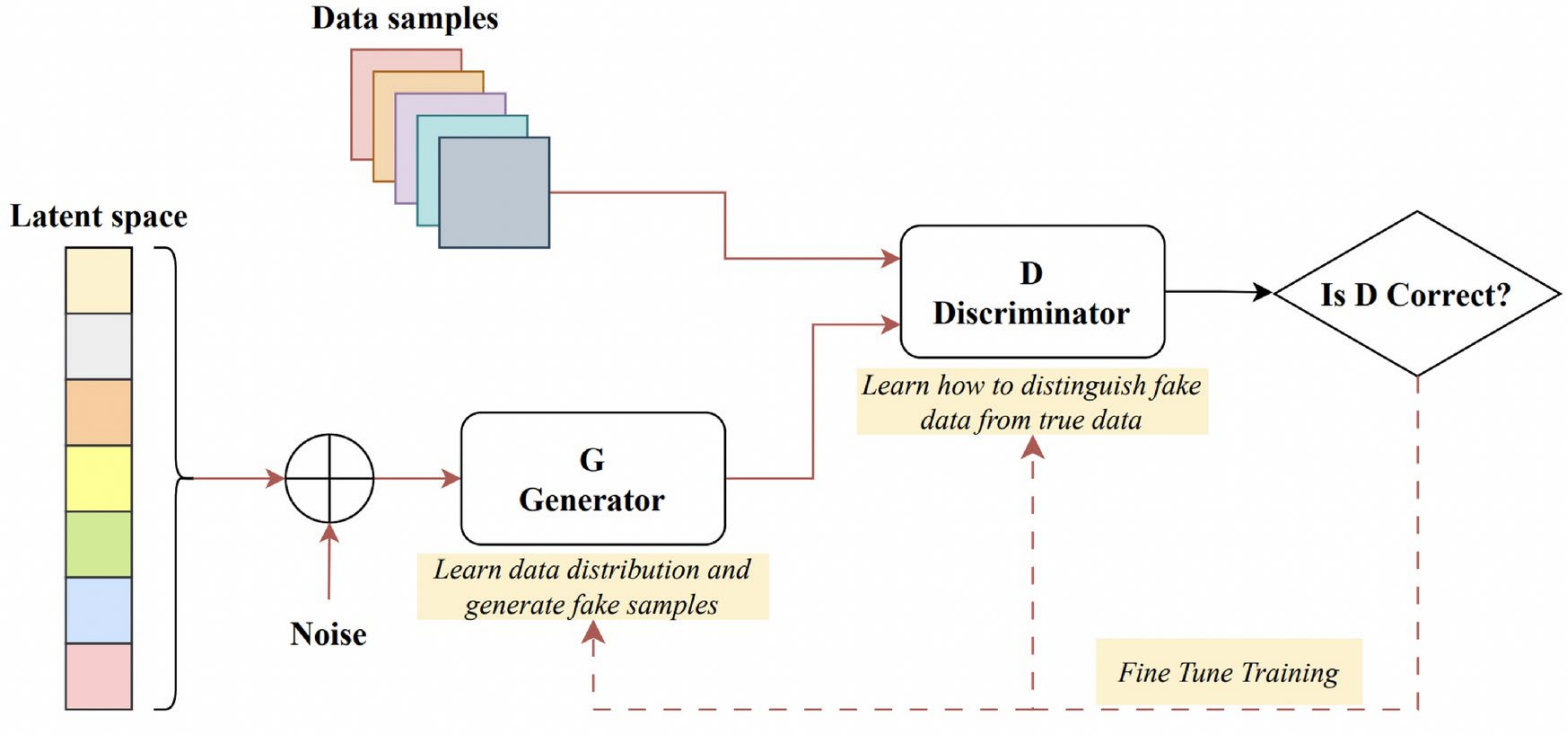
A sample generative adversarial network architecture—modified after^117^.

Goodfellow et al. introduced the Generative Adversarial Network (GAN) for synthesizing images from random noise^109^. A sample GAN architecture can be seen from Fig. 3. The generator crafts artificial images to deceive the discriminator, while the discriminator endeavours to distinguish genuine images from fabricated ones, a process termed “adversarial training”. This methodology can be adapted to train segmentation networks, where a generator is tasked with generating segmented images, and the discriminator differentiates between the predicted segmentation maps and the authentic ones. This modification prompts the segmentation network to yield more anatomically accurate segmentation maps^110,111^.

Son et al. introduced a technique employing generative adversarial training to create retinal vessel maps in the context of vascular segmentation. Their method enhanced the segmentation efficacy by employing binary cross-entropy loss during the training of the generator^112^. In 2020, K.B. Park et al. introduced a novel architecture called M-GAN, which aimed to enhance the accuracy and precision of retinal blood vessel segmentation by combining the conditional GAN with deep residual blocks^113^. The M-generator incorporated two deep FCNs interconnected through short-term skip and long-term residual connections, supplemented by a multi-kernel pooling block. This setup ensured the scale-invariance of vessel features across the dual-stacked FCNs. They integrated a set of redesigned loss functions to optimise performance, encompassing BCE, LS, and FN losses. Recently, Amran et al. introduced an adversarial DL-based model for automatic cerebrovascular vessel segmentation^114^. Their BV-GAN model utilised attention techniques, allowing the generator to focus on voxels more likely to contain vessels. This is achieved by leveraging latent space features derived from a prior vessel segmentation map, effectively addressing the issue of imbalance settings. In related research, Subramaniam et al. introduced a 3D GAN-based approach for cerebrovascular segmentation^115^. While their method used GANs for dataset augmentation (generating an extensive set of examples with self-supervision), Amran et al. aimed to enhance the U-NET-based segmentation directly through the GAN technique. Finally, earlier in 2023, Xie et al. introduced the MLP-GAN for brain vessel segmentation^116^. This method divided a 3D brain vessel image into three separate 2D views (sagittal, coronal, and axial) and processed each through distinct 2D conditional GANs. Every 2D generator incorporated a modified skip connection pattern integrated with the MLP-Mixer block. This design enhances the ability to grasp global details.

#### Vision transformers

**Fig. 4:**
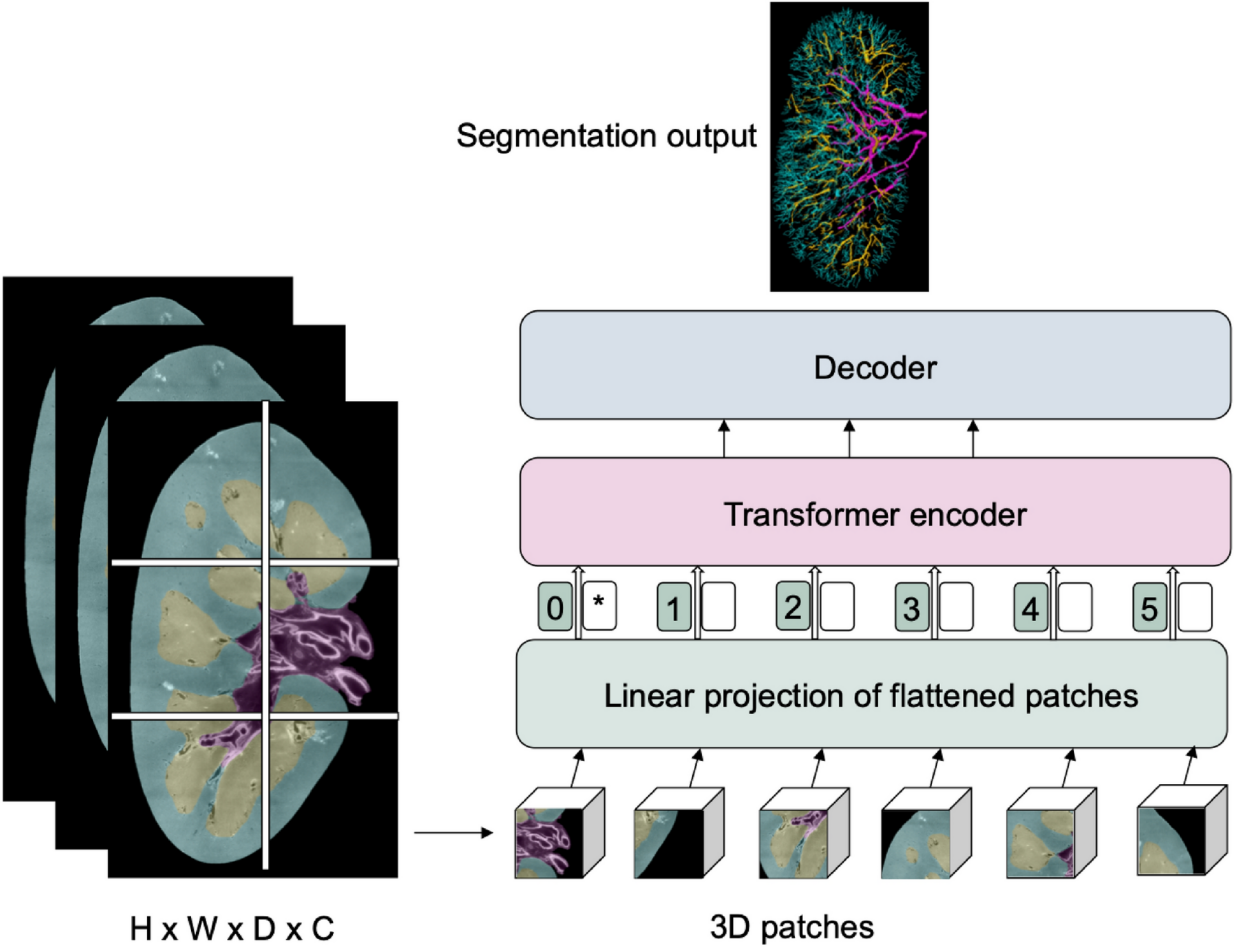
A sample vision transformer architecture for renal vasculature segmentation.

Transformers are primarily designed for sequence-to-sequence tasks but have shown significant promise in various domains, including computer vision. Vision Transformers (ViTs) are a type of neural network architectures that use transformer mechanisms to process images. Following their success in the realm of computer vision, researchers began to explore the potential of vision transformers for medical image segmentation tasks^118^. A sample vision transformer framework for vasculature segmentation is illustrated in Fig. 4. The input is a 3D medical image with dimensions H (height), W (width), D (depth), and C representing the number of channels (e.g., different modalities or scans). The image is divided into 3D patches, which are flattened and linearly projected into a lower-dimensional space before being passed into a Transformer encoder. The asterisk represents the extra [class] token, a learnable embedding added at the start of the patch sequence. This token interacts with all the patch embeddings within the transformer layers and eventually contributes to the final segmentation output through the decoder.

In 2021, Chen et al. came up with a model called TransU-Net, where a hybrid CNN-Transformer architecture is created to leverage both detailed high-resolution spatial information from CNN features and the global context encoded by Transformers by treating the image features as sequences^119^.

Pan et al. introduced the Cross Transformer Network (CTN), a new approach designed to understand 3D vessel features while considering their overall structure. CTN accomplishes this by combining the U-Net and transformer modules, allowing the U-Net to be more globally aware and better handle issues like disconnected or imprecise segmentation^120^.

Yu et al. introduced two new deep learning modules, called CAViT (Channel Attention Vision Transformer) and DAGC (Deep Adaptive Gamma Correction), to solve the problem of retinal vessel segmentation. CAViT combines two components: efficient channel attention (ECA) and the vision transformer (ViT). The ECA module examines how different parts of the image relate to each other, while the ViT identifies important edges and structures in the entire image. On the other hand, the DAGC module figures out the best gamma correction value for each input image. It does this by training a CNN model together with the segmentation network, ensuring that all retinal images have the same brightness and contrast settings^121^.

Zhang and his team, on the other hand, introduced a model named TiM-Net for efficient retinal vessel segmentation^122^. To capitalize on multiscale data, TiM-Net takes in multiscale images after maximum pooling as its inputs. Following this, they incorporated a dual-attention mechanism after the encoder to minimize the effects of noisy features. Concurrently, they utilised the MSA mechanism from the Transformer module for feature re-coding to grasp the extensive relationships within the fundus images. Finally, a weighted SideOut layer was created to complete the final segmentation.

The analysis of Tables 1, 2, 3 and 4 indicates a less frequent application of GANs and transformers (around 20%), suggesting that while U-Net and CNNs are the preferred methods in the field, there is still room for exploration and potential growth in the utilisation of GANs and transformers for vessel segmentation.

### Evaluation metrics

**Table 5:** Summary of Evaluation Metrics for Vascular Segmentation: TP = True Positive, TN = True Negative, FP = False Positive, FN = False Negative, $V_p$ = Predicted Volume, $V_g$ = Ground Truth Volume, *A*, *B* = Sets of Points for Spatial Distance, $T_{\text {prec}}$ = Topological Precision, $T_{\text {sens}}$ = Topological Sensitivity, *RI* = Rand Index, *x* = Predicted Segmentation Value, *y* = Ground Truth Value, *p*(*x*, *y*) = Joint Probability Distribution of *x* and *y*, *p*(*x*) = Marginal Probability Distribution of *x*, *p*(*y*) = Marginal Probability Distribution of *y*, *TPR* = True Positive Rate, *FPR* = False Positive Rate, *d*(*FPR*) = Differential of the False Positive Rate.

Metric category	Metric	Formula	Strengths	Limitations
Overlap-based	Dice Similarity Coefficient (DSC)	$DSC = \frac{2TP}{FP + FN + 2TP}$ ^123^	Easy to implement - widely used (26/31 studies). Good for overlap assessment.	Sensitive to class imbalance and lacks connectivity assessment.
	Sensitivity (Recall or TPR)	$Sensitivity = \frac{TP}{TP + FN}$ ^124^	Measures true positive rates.	Ignores structural/topological features.
	Specificity (TNR)	$Specificity = \frac{TN}{FP + TN}$ ^124^	Measures true negative rates.	Ignores structural/topological features.
	Precision	$Precision = \frac{TP}{TP + FP}$ ^124^	Accounts for false positives/negatives.	Rarely used in medical image segmentation.
	F1-Score	$F1 = 2 \cdot \frac{\text {Precision} \cdot \text {Recall}}{\text {Precision} + \text {Recall}}$ ^124^	Balances precision and recall.	Rarely used in medical image segmentation.
Topology-preserving	Centerline Dice (*clDice*)	$clDice = \frac{2T_{\text {prec}}T_{\text {sens}}}{T_{\text {prec}} + T_{\text {sens}}}$ ^125^	Preserves connectivity of thin, tubular structures like vessels.	Computationally expensive - less commonly used (2/31 studies).
Volume-based	Relative Volume Difference (RVD)	$RVD = \frac{|V_p - V_g|}{V_g}$ ^123^	Quantifies the disparity in volume.	Sensitive to small structures
Spatial distance-based	95th Percentile Hausdorff Distance (95HD)	*percentile*(*h*(*A*, *B*), 95)^126^	Captures boundary accuracy.	Sensitive to outliers or small discrepancies.
	Average Symmetric Surface Distance (ASSD)	$ASSD = \frac{1}{2} \left( \frac{1}{|A|} \sum _{a \in A} \min _{b \in B} d(a,b) + \frac{1}{|B|} \sum _{b \in B} \min _{a \in A} d(b,a) \right)$ ^127^	Provides average boundary accuracy.	Less sensitive to topology, focuses on boundary alignment.
Probabilistic-based	Area Under the Curve (AUC)	$AUC = \int _0^1 TPR(FPR) \, d(FPR)$ ^128^	Common in binary classification models, reflects overall performance.	Rarely used (3/31 studies).
Pair-counting-based	Adjusted Rand Index (ARI)	$ARI = \frac{\text {RI} - \text {Expected\_RI}}{\text {Max\_RI} - \text {Expected\_RI}}$ ^128^	Adjusts for chance in clustering assessments.	Not widely used in vascular segmentation.
Information-theoretic	Mutual Information (MI)	$MI(X,Y) = \sum _{x \in X} \sum _{y \in Y} p(x,y) \log \left( \frac{p(x,y)}{p(x)p(y)} \right)$ ^128^	Captures shared info between segmented and true data.	Complex to compute and rarely used in vascular segmentation.

**Fig. 5:**
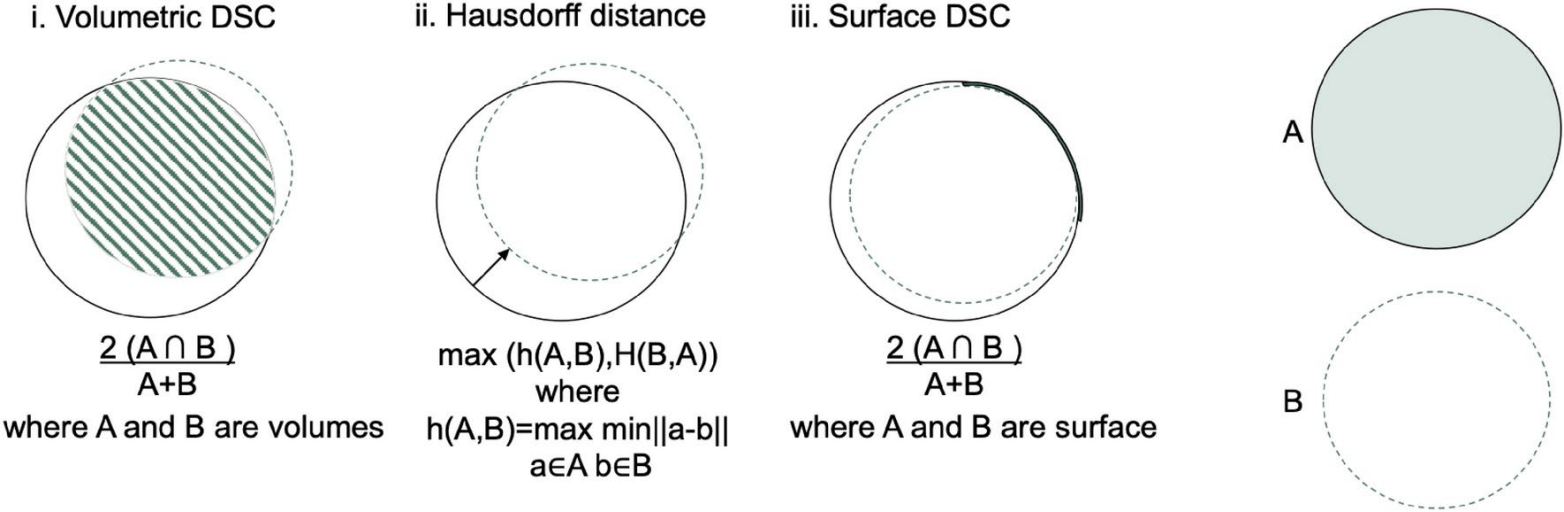
Illustration for calculation of volumetric Dice score coefficient (DSC), Hausdorff distance, and surface DSC. The solid line represents the ground truth contour, whereas the dashed line is the prediction. i. A Volumetric DSC, defined as the union of two volumes (green volume region) normalised by the mean of the two volumes. ii. Hausdorff distance, defined as the maximum nearest neighbor Euclidean distance (the arrow). iii. Surface DSC, is defined as the union of two contours (yellow contour region) normalised by the mean surface of the two contours.—modified after^129^.

In order to evaluate any automated vessel segmentation model or algorithm, a quantitative assessment of a segmentation task can be done through various performance metrics. Depending on the theory behind them, these metrics can be divided into overlap-based, volume-based, pair-counting-based, information-theoretic-based, probabilistic-based, and spatial distance-based measures^39^. Three commonly used metrics and their calculations are illustrated in Fig. 5. Note that the volumetric and surface DSC are identical, but applied to volume and surface area, respectively.

When assessing the performance of segmentation algorithms in comparison to ground truth, a contingency table is often employed, featuring True Positive (TP), True Negative (TN), False Negative (FN), and False Positive (FP) values. In this context, positive and negative denote pixels that, according to the ground truth segmentation, are attributed to vessels and background respectively.

A review of the literature reveals varied metric reporting across studies (as evidenced in Tables 1, 2, 3 and 4). This section delves into the most frequently used performance metrics, elucidating their underlying theory, strengths, and weaknesses.

*Overlap-Based Measures* Overlap-based measures are used to evaluate the similarity between the segmented result and the ground truth based on their overlapping regions. Such measures are the most widely utilised metrics with all studies we reviewed reporting at least one overlap based metric. However they have some limitations when applied to vascular segmentation which will be discussed further. The DSC is one of the most common overlap-based metrics that calculates the spatial overlap of two segmentations, with 26 of the 31 studies reviewed utilised DSC (see Tables 1, 2, 3 and 4). Given its properties, DSC is particularly used in vascular segmentation where the vasculature accounts for a small fraction (e.g. around 3% in the brain) of the organ volume, leading to unbalanced data challenges^130^.

Sensitivity, also known as TP Rate or Recall, is another overlap-based metric. It measures how well a model identifies TPs (e.g., correctly segmented vessels). It provides insight into how much of the ground truth overlaps with the predicted positive segment. Specificity, on the other hand, measures the proportion of TNs (e.g., correctly identified non-vessel areas) that are correctly detected. It reflects on how much of the ground truth’s negative area overlaps with the predicted negative segment. The calculations for these two measures, along with other metrics, are provided in Table 5.

Another related measure is precision, often referred to as the positive predictive value (PPV). While not frequently utilised in medical image validation, it plays a role in determining the F-Measure. The calculation for precision is presented in Table 5, while the F-Measure, also known as the F1 score, is the harmonic mean of precision and recall.

In 2020, Shit et al. developed centerline DSC (*clDice*), a unique topology-preserving loss function for tubular structure segmentation, as another DSC modification^125^. Their work highlighted that DSC metric does not necessarily assess the connectedness of a segmentation because DSC does not equally weight tubular structures with large, medium, and small radii. As connectivity is a crucial biological feature of vascular networks, bespoke metrics should be used to evaluate vascular segmentations. The authors demonstrated that training on a globally averaged loss causes a considerable bias towards the volumetric segmentation of big arteries in real vascular datasets compared to arterioles and capillaries (radius ranges e.g., 30 μm for arterioles and 5 μm for capillaries). To enable topology preservation, centerline DSC (*clDice*) is formulated using topological precision ($T_{\text {prec}}$) and topological sensitivity ($T_{\text {sens}}$). The formula for $clDice$ is provided in Table 5, while $T_{\text {prec}}$ and $T_{\text {sens}}$ are calculated as follows:

1 $$\begin{aligned} T_{\text {prec}} = \frac{\mid S_{P} \cap V_{L} \mid }{\mid S_{P} \mid } \text{, } \end{aligned}$$

2 $$\begin{aligned} T_{\text {sens}} = \frac{\mid S_{L} \cap V_{P} \mid }{\mid S_{L} \mid } \text{, } \end{aligned}$$

here $V_{L}$ is the ground truth mask, $V_{P}$ is the predicted segmentation mask, and $S_{P}$ (Predicted Skeleton) and $S_{L}$ (Labeled Skeleton) represent the skeletons extracted from the predicted and ground truth segmentations, respectively.

*Volume-based measures* These metrics focus on the volume or size of the segmented structures. As the name implies, Relative Volume Difference (RVD) is a typical volume-based metric that is based on the absolute difference in volumes of the segmented structure and the ground truth, usually normalised by the ground truth volume. It should be noted that RVD does not account for the spatial agreement between segmentations, as metrics like Dice would. For example, two objects of the same volume may receive a perfect score even if their shapes differ significantly, such as a square and a tree. Therefore, RVD fails to capture spatial correctness and should be complemented with metrics that assess spatial overlap and shape similarity, such as the Dice coefficient, to provide a more comprehensive evaluation of segmentation performance.

*Spatial distance-based measures*

Spatial distance-based measures focus on the spatial discrepancies between the segmented structures and the ground truth. For instance, the 95th Percentile Hausdorff Distance (95HD) assesses the greatest distance between a point in the true segmentation and the nearest point in the segmented result (Table 5). More formally, if we define $h(A, B)$ as the set of all distances from points in set $A$ to their nearest points in set $B$, then the 95HD is calculated as the 95th percentile of this set of distances. This measures the largest error in spatial alignment between the two sets, but by focusing on the 95th percentile, it avoids sensitivity to extreme outliers.

The Average Hausdorff Distance (AHD) is another commonly used metric in image segmentation and shape analysis to measure the similarity between two surfaces, typically the predicted segmentation and the ground truth (Table 5). It is derived from the Hausdorff Distance (HD), which captures the maximum distance between any point on one surface to the closest point on the other surface. The AHD refines this concept by averaging the distances between all points on the surfaces, making it a more robust measure than the standard Hausdorff Distance, which only considers the single greatest distance. Smaller AHD values indicate that the surfaces are more similar, with lower distances between corresponding points, while larger values reflect greater deviations in the segmentation’s boundaries.

Boundary-based techniques are another important subcategory of distance-based measures^131^. Normalised surface DSC, mean average surface distance, and average symmetric surface distance (ASSD) are three key metrics, especially in the field of vessel segmentation. Surface DSC is a measurement to evaluate the similarity between the segmented surface and a ground truth surface. Normalised surface DSC takes this a step further by normalising the measurement and making it less sensitive to size differences between the predicted segmentation and the ground truth. This is particularly useful for segmenting smaller structures, or size variability between subjects.

Surface distance measures the shortest distance of each point on the surface of the segmented structure to the surface of the ground truth. The ASSD is the average of these distances, calculated symmetrically between the two surfaces (Table 5). Specifically, the distance from each point on surface $A$ to the nearest point on surface $B$ is averaged, and the same is done for points on $B$ with respect to $A$, ensuring a balanced comparison between the surfaces.

The use of the appropriate assessment metric is crucial, as each measurement has specific biases dependent on the characteristics of the segmented structures. Whilst our review of literature shows that DCS or other generalised overlap measures are still the most frequently used metrics, more specialised vascular specific metrics are available and are becoming more widely implemented. The evaluation metric can strongly influence the choice of the final optimal model and hence should be chosen in accordance with the segmentation task’s nature and taking into consideration what downstream analyses are to be conducted on the segmentations, or the biological implications of a particular metric. For vascular structures, quantitative analyses of network topology for flow simulations are common end goals for segmentation^3,21^. In such cases meshing or skeletonising are common steps after segmentation. Meshing or skeletonisation algorithms are often highly sensitive to holes or cavities in the segmentations, or to breaks in connectivity in vascular components^132^. Thus, evaluation methods highly sensitive for connectivity breaks (e.g. clDice) should be implemented in these cases.

*Probabilistic-Based Measures*

Probabilistic-based measures evaluate the performance of segmentation and classification algorithms based on predicted probabilities rather than strict binary or discrete decisions. In binary classification, for example, a probabilistic model may assign a probability indicating the likelihood that an item belongs to the positive class rather than simply categorizing it as positive or negative.

The ROC curve (Receiver Operating Characteristic) stands as a prime example of a probabilistic-based measure. It graphs the sensitivity against (1-Specificity = 1-TNR = FPR) over a spectrum of decision thresholds. This curve offers a holistic perspective on a model’s capability to distinguish between classes.

The Area Under the Curve (AUC) encapsulates the model’s overall discriminative ability between positive and negative classes. From a mathematical standpoint, the AUC represents the integral of the ROC curve, which plots the True Positive Rate (TPR) against the False Positive Rate (FPR).

This reflects the model’s ability to distinguish between classes at various threshold settings. AUC appears in 3 of the 31 models reviewed in Tables 1, 2, 3 and 4.

Details on the pair-counting-based and information-theoretic-based metrics can be found in the Supplementary Material, as these metrics are not commonly employed in vessel segmentation.

## nnU-Net for kidney vessel segmentation

From the above review of the literature, we concluded that segmentation of blood vessels from HiP-CT data should be initially attempted using nnU-Net^107^. As nnU-Net is one of the most widely utilised frameworks for vessel segmentation tasks and has consistently delivered impressive results, we felt that this would provide the initial baseline against which any future development of more HiP-CT-specific frameworks should be benchmarked. Therefore, we prepared a training dataset from three different human kidneys, imaged with HiP-CT and semi-manually segmented by expert annotators. The goal was to assess how much training data is needed to provide adequate segmentation for a single kidney but also to investigate if nnU-Net trained on a subset of kidney data would generalise to a new kidney dataset given the high inter-sample variability seen between human organs.

In this section, we will provide a description of the HiP-CT kidney datasets, including its acquisition protocol and the segmentation process (“Hierarchical phase-contrast tomography (HiP-CT) kidney dataset” Section). Following that, we will present detailed information about the employed nnU-Net framework configuration (“nnU-Net framework” Section), and finally, we will present the results for nnU-Net application to kidney vascular segmentation of HiP-CT data “Evaluation” Section.

### Hierarchical phase-contrast tomography (HiP-CT) kidney dataset

**Table 6:** The size of the kidney volumes from the dataset used in the experiments together with their gender and resolution information.

Donor identifier	Experiment name	Dataset size (x, y, z) (pixels)	Gender	Age	Scanning voxel size (um)	Scan energy (keV)	Binned voxel size (um)
LADAF-2021-17	Kidney 1	1303, 912, 2279	M	63	25.0	81	50.0
S20-28	Kidney 2	1041, 1511, 2217	M	84	25.0	88	50.0
LADAF-2020-27	Kidney 3	1706, 1510, 501	F	94	25.08	93	50.16

Three human kidneys were used to create the training dataset - termed kidney 1, kidney 2, and kidney 3. Kidney 1 and kidney 3, were collected from donors who consented to body donation to the Laboratoire d’Anatomie des Alpes Françaises before death. Kidney 2 was obtained after a clinical autopsy at the Hannover Institute of Pathology at Medizinische Hochschule, Hannover (Ethics vote no. 9621 BO K 2021). The transport and imaging protocols were approved by the Health Research Authority and Integrated Research Application System (HRA and IRAS) (200429) and the French Health Ministry. Post-mortem study was conducted according to Quality Appraisal for Cadaveric Studies scale recommendations^133^. Sample preparation, scanning and tomographic reconstruction protocols are described in the references^16,21,82,134^. Basic scan parameters and demographic information are provided in Table 6

**Fig. 6:**
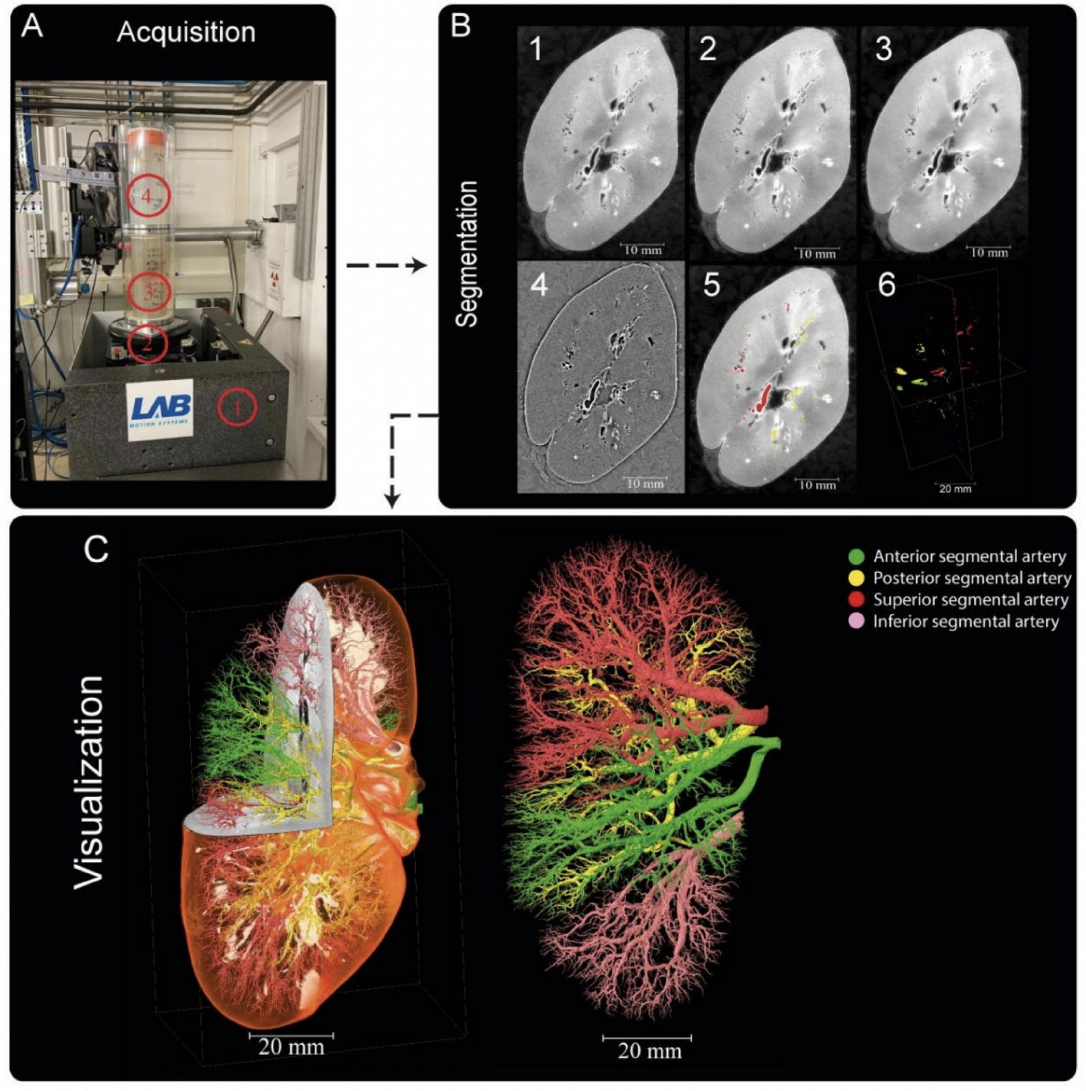
Processing pipeline for HiP-CT imaging and segmentation of human kidney vasculature. (**A**) Setup for imaging acquisition using HiP-CT at BM05; (1) tomographic stage, (2) platform, (3) sample, (4) reference sample (**B**) Image processing pipeline; (1) a 2D reconstructed image at 25 μmm3/voxel resolution; (2) binning the image by (2, 3) applying 3D median filter to increase signal-to-noise ratio, (4) Image normalisation using background detection correction, (5) Segmentation and thresholding, (6) Labelling the four main arterial branches (**C**) 3D rendering of the segmented vascular network of a human kidney. Each of the main four branching of the renal artery entering the kidney are colour-coded, Figure after^21^.

The annotation process of the three kidneys was carried out in Amira Version 2021.1. The reconstructed raw image data Fig. 6 B1 first underwent average binning x 2 Fig. 6 B2 from the acquired resolution ( ca. 25 μm) to ca. 50 μm, 3D median filtering was applied in Amira-Avizo v2021.1 (3 iterations 26 voxel neighbourhood) Fig. 6 B3 and filtering to visually enhance vessels appearance performed using background detection correction (Amira v2021.1; default parameter settings) Fig. 6B4. The annotation was conducted in a semi-manual fashion using the Amira v2021.1 magic wand tool. This is an interactive 3D region growing tool. Using this tool, annotators select a seed voxel within a vessel in a slice (slices can be in any one of three orthogonal directions); in addition, the annotator selects and refines a combination of intensity threshold, contrast threshold and hard-drawn limits, which are used to specify the stopping criteria of the 3D region growing. In some cases, it is necessary to manually draw using a paintbrush tool in slice-by-slice locations where vessels are infilled with blood or have largely collapsed. To provide assurance for labeling quality, an expert annotation validation process was implemented. 3D annotations were performed by a first expert annotator, after they were complete a second independent annotator conducted a 3D proofreading of these binary labels adding additional missing vessel labels again using three orthogonal planes for segmentation. After this second annotation round, five randomly selected 2D circular regions of the image were selected, and a third annotator marked all the labeled regions (one mark per region, which are the cross sections of the vessels). The third annotator then counted the True Positive (correctly segmented vessels), and False Negative (missed vessels), (note false positives are rare and easily removed due to the connected nature of the arterial tree). This provides a metric for what proportion of vessels have been traced or missed, and by sampling random areas in the dataset, directs the first two annotators to regions that may be poorly segmented. This triple validation process was iterated to achieve final annotations which can be accessed via^135^. It should be noted that vessels with diameters as small as 1-2 pixels could be segmented with the binning, median filter, and manual approach. Given the resolution of the images and their location within the generation vascular tree, these vessels represent interlobular arteries within the renal vascular tree and have been estimated from higher resolution HiP-CT of the same kidney, to be two branching generations from the kidney glomeruli (where the capillary bed is found). It is also of note that these samples come from a diverse age grouping 63-94yr old and have two males and one female represented. Finally, it is also worthy of note that in the case of kidney 2 (S20-28), the donor’s cause of death was COVID-19 which can cause microthrombi in the renal vasculature, and is seen as hyperintensities, particularly around the edge of the kidney context, this can be seen in Supplementary movies (1-3) which show slice by slice views of each kidney.

The number of slices and size of each slice segmentation were as followed (Z, X, Y): kidney 1: (2279, 1303, 912); kidney 2: (2217, 1041, 1511); and kidney 3: (501, 1706, 1510). The network topologies automatically generated for 3D full resolution configuration for experiment 1 were a patch size (Z, X, Y)of [192,112,112], a batch size of 2 and the number of pool per axis (Z, X, Y) were [5,4,4].

The imaging datasets used in this study are publicly available through the Human Organ Atlas data portal, where they can be accessed and visualised at https://human-organ-atlas.esrf.eu. Additionally, a Kidney Vessel Segmentation Challenge has been organised to facilitate the development of new deep learning methods for 3D blood vessel segmentation using HiP-CT data, with details available on Kaggle at https://www.kaggle.com/competitions/blood-vessel-segmentation/.

### nnU-Net framework

*Preprocessing*. Image preprocessing is a necessary step in machine learning, especially for image segmentation tasks. Notably, this step is crucial for enhancing model performance, generalization, and robustness when handling real-world data. nnU-Net incorporates a comprehensive set of automated preprocessing steps within its data preparation pipeline. These steps include:

- *Data Augmentation*: Rotations, scaling, Gaussian noise, Gaussian blur, brightness and contrast adjustments, simulation of low resolution, gamma correction, and mirroring.
- *Intensity Normalization*: Global dataset percentile clipping, z-score normalization with a global foreground mean, and z-score normalization on a per-image basis.
- *Resampling Strategies*: In-plane resampling using third-order spline interpolation and out-of-plane resampling with either nearest neighbor or third-order spline interpolation.
- *Image Target Spacing*: Determined based on the lowest resolution axis tenth percentile, axes median, and median spacing for each axis.

During preprocessing, nnU-Net automatically extracts a dataset fingerprint, which encompasses dataset-specific properties such as image sizes, voxel spacings, and intensity information. It also computes relevant dataset statistics and configures the training parameters based on both the dataset characteristics and the available computational resources. As a result, parameters like batch size and other configuration settings are automatically determined by nnU-Net and used directly in the experiments. For a detailed overview of the automated method configuration, refer to Supplementary Fig. 1^107^.

*Architecture*. It is important to mention that the nnU-Net framework does not have a new deep learning architecture. However, it covers the U-Net family, which has encoder-decoder architectures, including 2D U-Net^88^, 3D U-Net^95^, and Cascaded 3D U-Net^136^. It also provides an ensemble option, which explores 2D U-Net, 3D U-Net, or 3D cascade results and chooses the best model (or combination of two) according to cross-validation performance.

*Postprocessing*. The nnU-Net framework also provides optional configuration for postprocessing on the full set of training data and annotations, including treating all foreground classes as one individual class (depending on the largest component suppression increases in cross-validation performance)

*Configuration and Training Process*. We trained the nnU-Net framework using the 3D U-Net version with 3-fold cross-validation implemented on the training sets. The evaluation results presented in the following section are the averages on testing sets of 3-fold cross-validation. We used the nnU-Net default auto-generated hyper-parameters for training, which include the learning rate^91^ initialised as 0.01 with a polynominal decay policy of $(1 - epoch/epoch_{max})^{0.9}$, the loss function as the sum of cross-entropy and DSC loss^137^, an optimizer based on stochastic gradient descent (SGD) with Nesterov momentum value of 0.99 and epoch number of 1000 with mini-batches of 250. Notably, the default nnU-Net typically requires $>30$ million parameters for the training. The proposed model was implemented in Python language^1^ using Pytorch^138^. All experiments were on an NVIDIA TITAN RTX 24GB GPU. The training processes for Experiments 1, 2, and 3 are shown in Supplementary Figs. 2, 3 and 4, respectively.

**Fig. 7:**
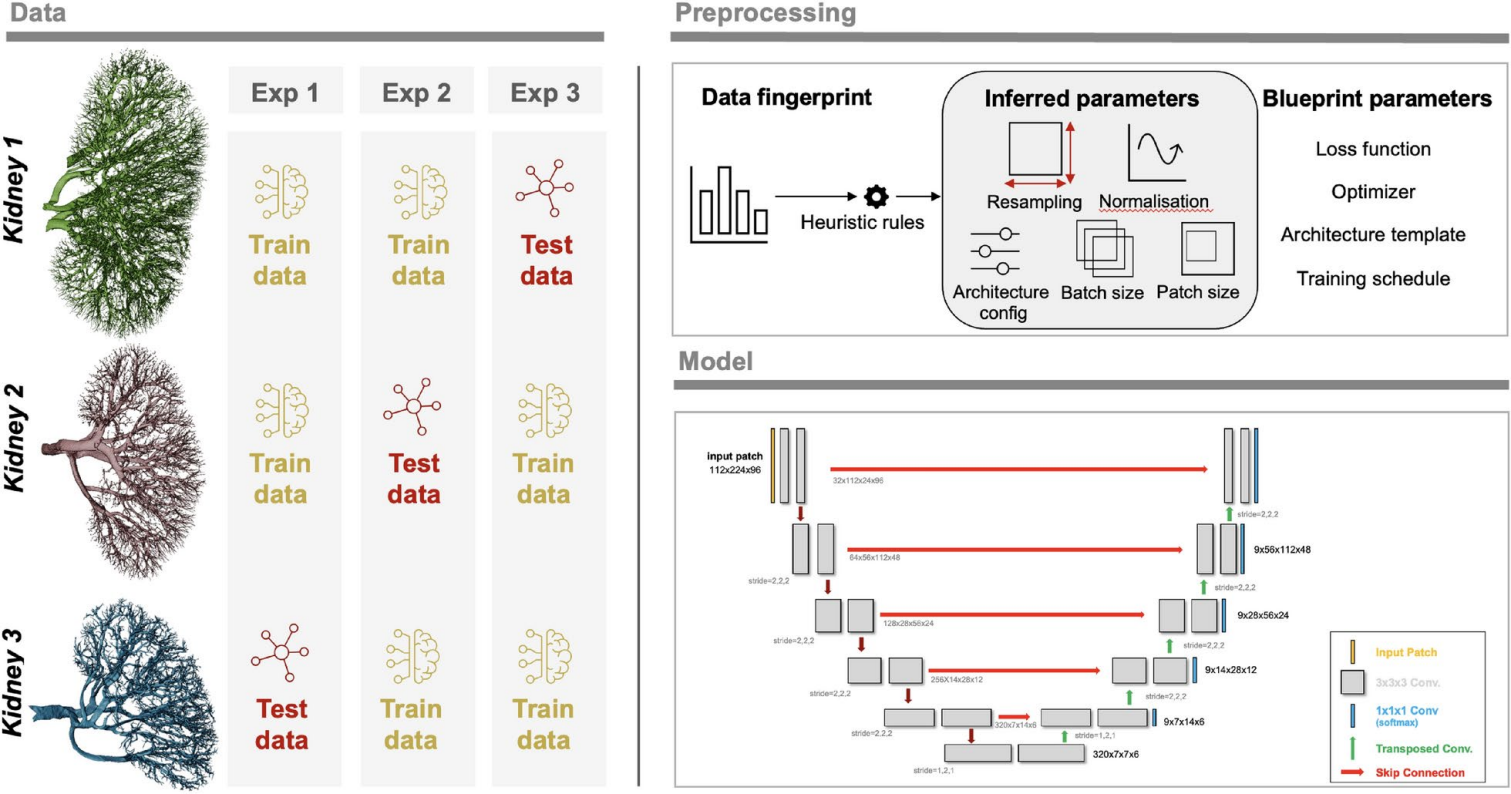
Overview of the experiments together with a sample 3D nnU-Net architecture.

### Evaluation

We chose to perform four experiments. In three experiments, we used two of the kidneys as training data and one as test data. These aimed to investigate how well the nnU-Net approach would generalise to a new dataset where the donor and thus anatomy and sample prep may differ slightly; it also had the virtue of testing how different-sized training datasets impacted nnU-Net training output. The overview of the experiments can be seen in Fig. 7.

The fourth experiment aimed to see how well nnU-Net could segment the remainder of a dataset given a limited amount of labeled slices from the same dataset. Due to the size of HiP-CT datasets, such a model still has utility as segmenting vascular networks for entire organs is highly time-consuming^21^. In this section, we selected kidney 1 for our experimental evaluation. Half of the kidney has been utilised as a training set (1139 slices), and the remaining half as a testing set (1140 slices). The kidney is divided into two symmetrical halves horizontally (when the kidney figures shown in the manuscript are considered), ensuring that both sides contain a similar distribution of large and small vessels. Please note that the purpose of this experiment is to determine whether the annotation process can be accelerated by annotating only half of the kidney. As a result, it inherently differs from the other experiments and does involve some degree of data leakage.

The importance of evaluation metrics in automatic image processing with machine learning cannot be emphasised enough, as they serve as a basis for determining the choice or practical applicability of a method. However, much of the research has been on creating novel image processing algorithms leaving the critical issue of reliably and objectively evaluating the performance of these algorithms largely unexplored^131^. Moreover, some of the commonly used evaluation metrics do not always correlate with clinical applicability^129,139^, and the specific features of a biomedical problem may make certain metrics unsuitable, such as when the DSC is utilised to evaluate extremely small structures^140^. As a result, carefully choosing the right evaluation metric for a given problem becomes important for validating and comparing the performance of image processing methods.

In vessel segmentation, class imbalance presents a significant challenge where the pixel count for the foreground class (vasculature) is notably lesser than that of the background class (non-vasculature).

Class imbalance can hinder effective network training, as most data points are typically negative samples (or backgrounds) that often do not offer valuable learning insights. Moreover, these negative samples can dominate the positive ones (such as arteries) during the training process as loss values are predominantly generated from the negative samples.

**Table 7:** Results of all four experiments done using nnU-Net.

Expt.	Train data	Test data	DSC	CLD	NSD (t = 1)	NSD (t = 0)	ASSD
1	Kidney 1,2	Kidney 3	0.9410	0.8886	0.9651	0.7631	4.300
2	Kidney 1,3	Kidney 2	0.9523	0.8533	0.9518	0.7120	0.8639
3	Kidney 2,3	Kidney 1	0.8585	0.8228	0.8968	0.7132	2.9270
4	Half of kidney 1	Other half of kidney 1	0.9513	0.8631	0.9404	0.8549	2.1561

*SD* surface distance, *NSD* normalised surface DSC, *CLD* centerline DSC, *t* tolerance (in voxels), *ASSD* average symmetric surface distance

**Fig. 8:**
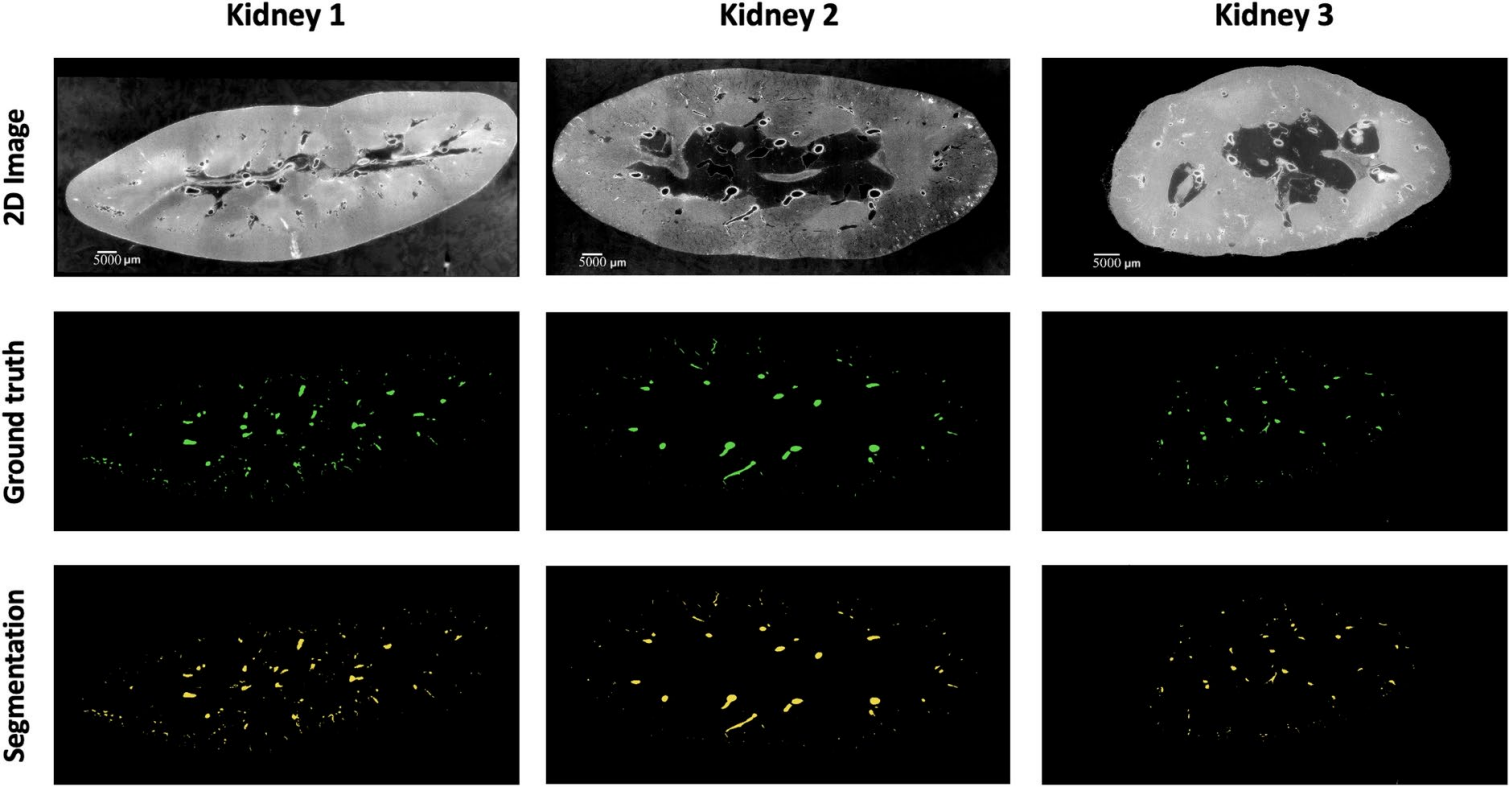
A sample 2D slice from each kidney together with their corresponding ground truth labels and the output of the model segmentations (for kidney 1 the segmentation is provided by Expt. 3).

A combination of overlap-based and boundary-based metrics is selected to evaluate different properties of the model predictions^131^. For overlap-based metrics, DSC is computed and is complemented by the Centerline DSC (clDice)^125^ due to the tubular nature of blood vessels and the importance of connectivity for vascular structures. Overlap-based metrics have certain limitations such as shape unawareness and inaccurate assessment when dealing with small structures hence, boundary-based metrics, specifically Normalised Surface DSC (NSD) and Average Symmetric Surface Distance (ASSD), are also computed using MONAI v1.2.0^141^. The results of the four experiments are provided in Table 7 and representative images for each kidney shown in Fig. 8.

## Results

In this study, we evaluated model performance for the segmentation of the arterial network from HiP-CT images of whole human kidney. Five metrics: DSC, clDice, NSD (t=1), NSD (t=0), and ASSD, with results presented in Table 7 were used to evaluate the outputs. The range of metrics we employed in our experiments provides a thorough overview of model performance and allows the state-of-the-art presented to be rigorously benchmarked against future research. For each experiment, one unseen kidney is selected as test data for evaluation of the model’s generalisation capability. The 5 metrics are calculated on test data after the model training is finished. Beyond quantitative evaluations, we provided visual insights through 3D representations of the ground truth, model predictions, and false negatives.

**Fig. 9:**
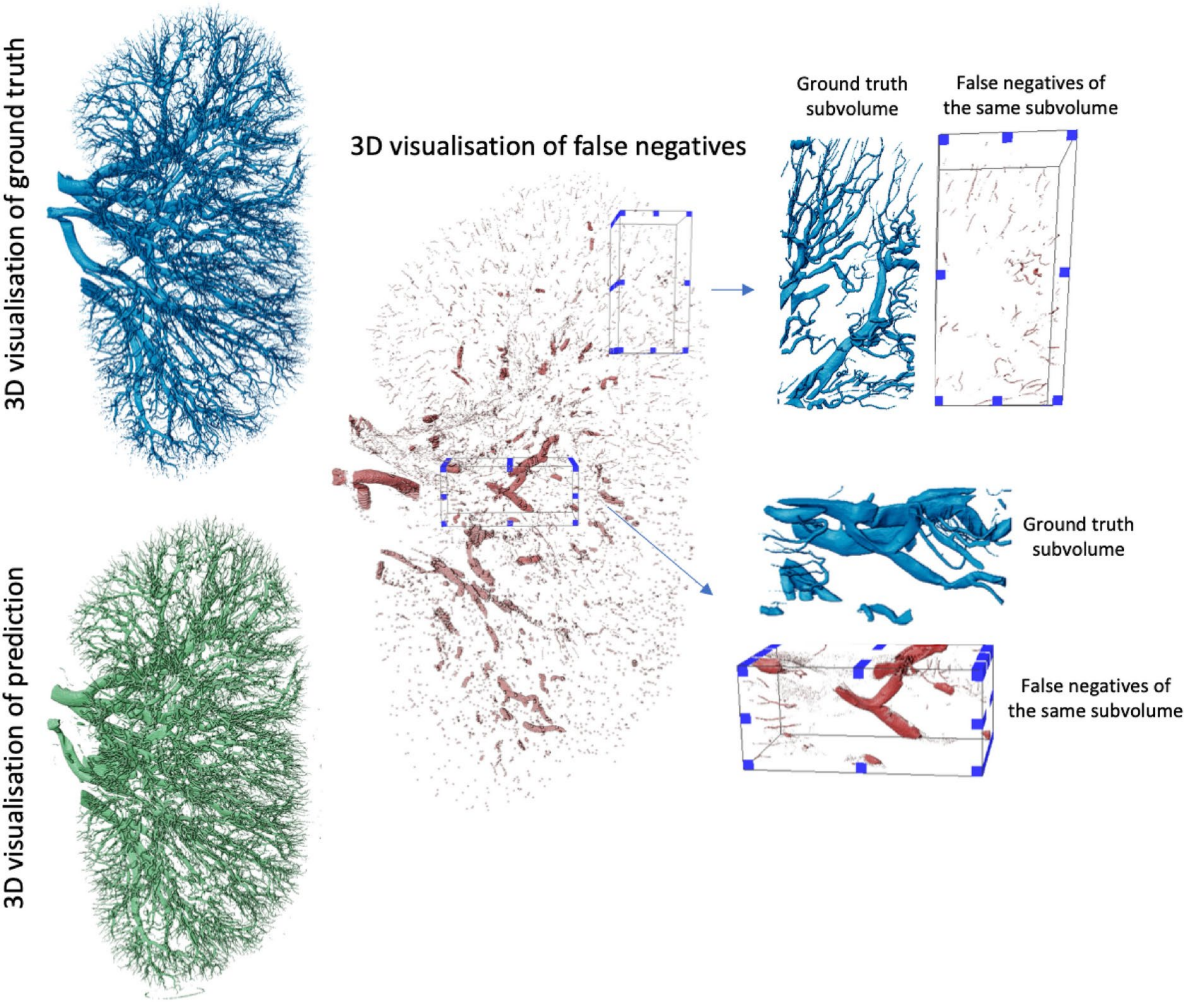
The visualisation of kidney 1 predictions from Experiment 3 showing the model’s false negative prediction together with subvolumes taken from it indicating the regions where model did perform worst.

Based on our literature review (see Tables 1, 2, 3 and 4), it is clear that the DSC, also known as Dice, is the predominant metric for evaluating vascular segmentation model performance. Therefore, employing DSC for result evaluation is essential to align with existing literature, such as in^50^. This metric quantifies the overlap between segmentation predictions and ground truth. In our three experiments, the first and second experiments yielded superior segmentation results on the unseen kidney, with DSC of 0.9523 and 0.9410, respectively, whereas the third experiment attained only 0.8585. 3D examination of kidney 1, as shown in Fig. 9; revealed more collapsed vessels compared to kidney 2 and 3, potentially explaining the lower DSC when the first kidney was the test subject. Nevertheless, DSC primarily assesses voxel-to-voxel concordance, overlooking several crucial characteristics of the vessels. Hence, it should not be the sole metric for deciding the performance of vascular segmentation.

In “Evaluation” Section, we describe an additional experiment (experiment four) to evaluate the nnU-Net’s performance with a limited dataset. In this case we used just kidney 1. We trained the model on half the data and tested it on the remaining half. This experiment’s training-test division was designed to test the model’s capabilities, to extrapolate from a small subset of images within a single organ to the entire dataset. This reduces the challenge of the segmentation task but also reduces the utility of the final model, however given the size of HiP-CT datasets, such an approach is likely to still have utility in particularly challenging or anatomically unique datasets. The performance metrics for the test set were DSC = 0.9513, clDice = 0.8631, NSD (t=1) = 0.9404, NSD (t=0) = 0.8549, and ASSD = 2.1561. These results indicate that nnU-Net is effective at segmenting ’unseen’ kidney tissues from the same organ, reflecting its capability to learn kidney anatomy and thereby enhancing the efficiency of the labeling process. It also shows the expected improvement by comparison to the other three experiments, indicating the challenges with accounting for anatomical variation across human samples, or variations in the data aquisition and data reconstruction parameters see^16,134,142^ for details on protocol variations.

## Discussion

The nnU-Net framework demonstrated significant success in accurately segmenting kidney vessels. The results provide key insights into how nnU-Net can address the challenges posed by hierarchical, connected vascular networks, making it highly relevant for applications in high-resolution imaging, such as HiP-CT. Given the nature of vasculature networks - hierarchical connected networks of thin and elongated tubular structures, ensuring sensitivity to small as well as large vessels, mitigating boundary ambiguities and ensuring connectivity are all important factors. Therefore, we reported a number of different metrics, and provided qualitative outputs to understand what features each metric best represents. Distance metrics applied included the ASSD (see equation 15) and the NSD to evaluate the similarity of the segmented regions’ surfaces (or boundaries) rather than the volumetric overlap. It is particularly useful in scenarios where small boundary deviations can be critical, such as in segmenting thin structures or lesions. Tolerance (*t*) is an important hyper-parameter when it comes to NSD, which relates to the acceptable amount of deviation in the segmentation boundary in voxels. If a point on the boundary of the prediction is within this tolerance distance from a point on the boundary of the ground truth, it is considered a TP. Therefore, an appropriate NSD tolerance should usually be selected based on inter-annotator variability or some other heuristic. In our experiment, we initially set tolerance to a strict 0 voxels as for the smallest vessels the diameter can be as small as two voxels. We also calculated the value with 1 voxel tolerance, with the latter achieving around 25% increase in the NSD score. The choice of t=0 voxels for our primary experiment was driven by the small diameter of the kidney vessels, ensuring that even the smallest vessels were accurately assessed. For larger vessels, an increased tolerance would likely be acceptable without drastically impacting the performance assessment, as the proportional effect of the tolerance would be smaller relative to the vessel size. However, for the smaller vessels, any increase in tolerance could lead to an overestimation of the segmentation accuracy. This is why, in cases where vessels of various sizes are involved, using a metric with a variable tolerance based on the ground truth vessel radius could provide a more nuanced evaluation. Such a method would allow larger vessels to be evaluated with a higher tolerance and smaller vessels with a stricter one, potentially offering a more balanced assessment across varying vessel sizes.

Capturing vessel continuity is vital for vascular network analysis. To this end, we applied clDice (see equation 7), a metric specifically developed to be sensitive to leaks in the continuity of vessel centerline irrespective of vessel radius. This metric reduces bias toward algorithms that predict larger structures such as large arteries but miss microvasculature structures. The cldice scores across all experiments are lower than the DSC or NSD (t = 1) reflecting its lack of bias towards the larger vessels and sensitivity to discontinuity in small vessels as seen in Fig. 9. In addition, the ranking of outputs does not follow the same pattern when the cldice rather than the DSC, NSD or ASSD scores are considered, with Epxt. 1 having the highest score, and Expt. 3 the lowest. It should be noted that since clDice metric incorporates a morphological thinning approach to skeletonize i.e reduce the vessels to their centerlines, small holes or highly multi-scale vasculature such as that imaged by HiP-CT can result in highly irregular skeletons e.g. ball-like or star-like structures which impact the ability of cldice to provide a comparative metric (See Supplementary Fig. 5) data.

The introduction of the HiP-CT technique has brought both opportunities and challenges to vascular segmentation. While its high resolution enables detailed visualisation of intricate structures, this comes at the cost of significantly increased manual annotation time, which is both time-consuming and subjective. Also, while HiP-CT offers significant advantages over techniques like MRA and CTA, its application is currently restricted to research scenarios and cannot be used in clinical settings where in-vivo imaging is required. At this stage, it is also important to acknowledge that some of the challenges in detecting the smallest vessels may come from annotation. Human annotators were responsible for the labeling process of this dataset utilising a semi-manual, interactive 3D region growing tool. As with all data created by human annotators there is the likelihood that some of smallest vessels in these datasets were missed by the independent annotators. This highlights why methods which treat human-annotated data as a gold standard rather than a ground truth, can be so powerful in e.g. active learning frameworks^143^. Deep learning offers a valuable solution by automating the segmentation process, reducing annotation efforts, and improving consistency across datasets. By leveraging advanced algorithms, the need for manual intervention can be minimized, enhancing scalability and accuracy in segmenting complex vascular networks. Our study shows that nnU-Net successfully captures some of the small vessels missed by manual segmentation (see Supplementary Fig. 6).

It should be noted that the evaluation in this study was performed specifically on kidney vessels, which may limit the generalisability of the results to other types of vessels. Different anatomical structures, vessel sizes, and complexities in other organs could require adjustments in model or further validation to ensure applicability beyond kidney vessel segmentation. Also, another key limitation is that only the 3D nnU-Net framework was evaluated, which limits the ability to assess the performance of other segmentation models that might handle small, intricate structures differently. Additionally, inter-rater reliability assessment of the vessel segmentation was not performed, due to the time-consuming nature of manual segmentation at this high level of resolution and our approach for triple annotator verification and our approach for triple annotator verification.

From the discussion on metrics above, the benchmarking methodology of nnU-Net underperforms in several scenarios when applied to HiP-CT data on vascular segmentation. With whole organ HiP-CT we are able to manually segment and create training data down to the level of the interlobular arteries or approx. two generations from the capillary bed. These arteries have a minimum radius of ca. 40 μm^21^, (1–2 pixels in diameter). However, the experiment scores reveal that the failed prediction on such small vessels leads to discontinuity of the vascular network. The work in^125^ has shown an improved continuity performance on vasculature segmentation with clDice as part of the loss function when training the network, though this comes at a high computational cost and has challenges already highlighted with the skeletonisation of multi-scale structures. Integration of rich hierarchical representations of thin and long structures by use of spatial attention and channels attention modules could also potentially improve the outcomes^144^. Another challenge that may hinder vasculature continuity is edge identification and segmentation. Considering the imbalance of edge and non-edge voxels, an edge-reinforced neural network (ER-Net)^145^ combining a reverse edge attention module^57^ and an edge-enforced optimisation loss to discover spatial information of the edge structures could potentially increase the vasculature continuity on segmentation results. In both the small vessel and edge enhancement cases it is important to remember that HiP-CT is a propagation-based phase contrast technique and thus has intrinsically higher contrast for small thin structures and edges between structures. Tomographic reconstruction is a part of the process of HiP-CT data volume data production and can have a large impact on the ease of segmentation. Tuning the tomographic reconstruction pipeline to enhance specific features, whilst challenging could be a powerful approach to segmenting specific smaller structures.

Relative to the ground truth, the benchmark model appears to overlook certain vasculature, particularly struggling to identify certain large vessels as shown in Fig. 9. Upon closer examination of the pertinent sub-volumes, it becomes evident that these misdetected large vessels correspond to collapsed vessels within the organs. As shown in Fig. 9 of the prediction on kidney 1, collapsed vessels have a flat appearance, morphologically different from the noncollapsed vessels seen elsewhere. This suggests that the model’s performance falters notably in the presence of disrupted structures, and these could be the potential cause for the observed lower scores when kidney 1 is used as the test set. In future approaches, addressing the challenge of collapsed vessels could involve employing a data augmentation method. By applying transformations that accurately simulate the morphological changes observed in collapsed vessels, the algorithm’s ability to accurately segment collapsed vessels can be enhanced. Key transformations could include selectively compressing or flattening portions of the vessels in certain images. Additionally, adjusting the vessels’ shape while ensuring these modifications are medically realistic would be essential.

HiP-CT scans in high-resolution present challenges to deep learning methods applied to different tasks such as segmentation. These scans highlight more details that must be captured by the models. Using a larger patch size for training can ease the problem. However, there is a trade-off between the GPU memory and the size of the receptive field. As a result, integrating regional features and their global dependencies remains a research direction.

## Conclusion and future work

The vascular system is vital for nurturing and supporting every organ in the body, and its abnormalities can be indicative of various diseases. Developing accurate and automated segmentation of vasculature, that can be applied to nasent imaging technologies such as HiP-CT, could pave the way for scalable vascular segmentation across large-scale datasets. Scalability in turn enables quantification across a large demographic population, therefore assisting the construction of a data-driven Vascular Common Coordinate Framework^146,147^ for human atlasing projects such as the Human BioMolecular Atlas Program^148^, the Human Organ Atlas Project^149^ and the Cellular Senescence Network Program^150^.

This paper offers a comprehensive review of recent literature focusing on deep-learning techniques for blood vessel segmentation. We primarily delve into the recent deep-learning methodologies, emphasizing the challenges associated with vasculature segmentation. Our aim with this review is to lay a solid foundation for researchers, building robust models for vessel segmentation, especially using phase contrast imaging.

For these models to be widely accepted in the medical field, we need to address certain gaps in the current research. From our literature survey, we deduce the following:

(i) Publicly accessible datasets for vascular segmentation are limited. Even within private datasets, the size available for training is often limited.
(ii) Labeling the vascular region remains a formidable challenge and requires extensive effort.
(iii) Most studies employ private data and provide internal validation. This makes direct comparisons between studies challenging and prevents the assessment of algorithm efficacy.
(iv) Every phase of the imaging process; from sample preparation, imaging, modality specific artefact correction, pre-processing and algorithm optimisation to post-processing, holds immense importance. The post-processing stage, especially, has a great impact on the final segmentation^151,152^.

Many studies focus on mapping out complex blood vessel networks, mostly concentrating on larger vessels yet the significance of smaller vessels, which are frequently overlooked, cannot be understated from a (patho-)physiological point of view^19,153^. Moreover, the choice of evaluation metrics and loss functions in vessel segmentation is often unclear, calling for clearer guidelines. Through our study, we aim to assist researchers in identifying the most suitable metrics for their analyses. One significant challenge with vessel segmentation models is their limited generalisability, as most studies rely on private datasets. To enhance adaptability, models should be cross-validated against diverse data. Providing both more public datasets and pre-trained model weights could benefit future research. By making our data publicly accessible, through a Kaggle competition (https://www.kaggle.com/competitions/blood-vessel-segmentation/data) and following its conclusion, through the human organ atlas portal (www.human-organ-atlas.esrf.eu), we invite researchers to evaluate their algorithms on HiP-CT data, fostering advancements in the field.

## Supplementary Information

Supplementary Information.

## Data Availability

The datasets analysed in the present study are publicly accessible and available online: https://human-organ-atlas.esrf.eu/.

## Footnotes

1 https://www.python.org.
