# Supplementary material for: Deep learning for 3D vascular segmentation in hierarchical phase contrast tomography: a case study on kidney
Source: Sci Rep. 2024 Nov 8;14:27258. doi: 10.1038/s41598-024-77582-5 (PMC11549215; doi:10.1038/s41598-024-77582-5)
Supplement: Supplementary file 1 — Supplementary Information. [file 41598_2024_77582_MOESM1_ESM.pdf]

Supplementary Information for Deep Learning for 3D Vascular Segmentation in Hierarchical Phase Contrast Tomography: A Case Study on Kidney

Ekin Yagis[1],*, Shahab Aslani[1,4], Yashvardhan Jain[3], Yang Zhou[1], Shahrokh Rahmani[1,9], Joseph Brunet[1,2], Alexandre Bellier[5], Christopher Werlein[6], Maximilian Ackermann[7], Danny Jonigk[8], Paul Tafforeau[2],+, Peter D. Lee[1],+, and Claire Walsh[1],+

[1],*Department of Mechanical Engineering, University College London, London, UK

[2]European Synchrotron Radiation Facility, Grenoble, France

[3]Department of Intelligent Systems Engineering, Luddy School of Informatics, Computing, and Engineering, Indiana University, Bloomington, USA

[4]Centre for Medical Image Computing, University College London, London UK

[5]Laboratoire d'Anatomie Des Alpes Francaises, Grenoble, France

[6]Institute of Pathology, Hannover Medical School, Carl-Neuberg-Straße 1, 30625, Hannover, Germany

[7]Institute of Functional and Clinical Anatomy, University Medical Center of the Johannes Gutenberg-University Mainz, Mainz, Germany

[8]Member of the German Center for Lung Research (DZL), Biomedical Research in Endstage and Obstructive Lung Disease Hannover (BREATH), Hannover, Germany

[9]National Heart and Lung Institute, Faculty of Medicine, Imperial College London, London, UK

*e.yagis@ucl.ac.uk

+these authors contributed equally to this work

Supplementary Notes

Supplementary Note 1: Additional Information regarding GANs

GANs represent generative models designed to approximate real data distributions, enabling them to generate novel image samples. GAN models are commonly employed for tasks such as image-to-image translation (cross-modality synthesis), image synthesis, and data augmentation. Comprising two distinct networks, a generator and a discriminator, GANs function by pitting these networks against each other during training.Conditional GANs (cGANs), on the other hand, are a modified version where the generator creates images depending on specific conditions or inputs, which can be useful in vessel segmentation.

Supplementary Note 2: Additional Information regarding Vision Transformers

Instead of being processed by pixel values, images are segmented into setsized, non-overlapping sections, such as 16x16 pixels for medical image segmentation using ViTs. These sections are linearly transformed into singular vectors, a procedure called tokenization. Subsequently, to preserve the spatial context, positional embeddings are integrated with the tokenised patches. These enhanced embeddings navigate through various layers of the standard transformer encoder. To form a segmentation mask, a decoding method, which might be an upsampling layer or an alternative transformer, is applied to produce labels for each pixel in the image.

The Swin Transformer is a modified version of the Vision Transformer (ViT), designed to further adapt the transformer structure for image-related tasks and boost its efficiency. ``Swin" gets its name from ``Shifted Window," reflecting a key feature of its design. In July 2023, Wu and his team developed the Inductive BIased Multi-Head Attention Vessel Net (IBIMHAV-Net) [1]. The architecture is formed by extending the Swin Transformer to 3D and merging it with a potent mix of convolution and self-attention techniques. In their approach, they used voxel-based embedding instead of patch-based, to pinpoint exact liver vessel voxels, while also utilizing multi-scale convolution tools to capture detailed spatial information.

Supplementary Note 3: Additional Information regarding Metrics

Pair-Counting-Based Measures

The pair-counting-based measures are calculated based on the correspondence between object pairs in the segmentation and the ground truth. One such metric is the Adjusted Rand Index (ARI), which adjusts the Rand Index (RI) for the chance grouping of elements. The ARI is calculated as follows:

$$ARI = (RI - Expected\_RI) / (Max\_RI - Expected\_RI),$$

Where RI is the Rand Index, calculated as:

$$RI = (a + d) / (a + b + c + d),$$

In this formula, 'a' is the number of pairs of objects that are in the same group in both the predicted segmentation and the ground truth (corresponding to true positives, TPs), and 'd' is the number of pairs of objects that are in different groups in both (corresponding to true negatives, TNs). 'b' and 'c' correspond to false positives (FPs) and false negatives (FNs), respectively.

Information-Theoretic-Based Measures
As the name implies, information-theoretic-based measures use information theory concepts to estimate the quality and performance of the segmentation. For instance, one such measure called Mutual Information (MI) is calculated based on the shared information between the segmented result and the ground truth.

For two discrete random variables X (segmentation result) and Y (ground truth), the MI is defined as:

$$MI(X,Y) = \sum_{x \in X} \sum_{y \in Y} p(x,y) \log(p(x,y) / (p(x)p(y))),$$

Where:

- $p(x,y)$ is the joint probability distribution function of X and Y.

- $p(x)$ is the marginal probability distribution function of X.

- $p(y)$ is the marginal probability distribution function of Y.

X might represent the predicted segmentation, where a specific value x taken by X could be either 'object' (e.g., a vessel) or 'background.' Y represents the ground truth (actual segmentation), where a specific value y taken by Y could similarly be either 'object' or 'background.'

In that context:

- p(x='object', y='object') corresponds to the probability of a TP.

- p(x='background', y='background') corresponds to the probability of a TN.

- p(x='object', y='background') corresponds to the probability of a FP.

- p(x='background', y='object') corresponds to the probability of a FN.

Supplementary Tables

Supplementary Table 1: Details of preprocessing and training (3D_fullres) including the total number of training and evaluation data for each experiment and training time.

Exp.	Pre-processing	Training data	Evaluation data	Median image size in voxels	Batch size	Patch size	Training time
1	Normalisation: zscore	Kidney 1 (1303,912,2279) & Kidney 2 (1041,1511,2217)	Kidney 3 (1706x1510,501)	[1211.5, 1172.0, 2248.0]	2	[112, 112, 192]	35.27 hours
2	Normalisation: zscore	Kidney 1 (1303,912,2279) & Kidney 3 (1706x1510,501)	Kidney 2 (1041,1511,2217)	[1211.0, 1504.5, 1390.0]	2	[128, 128, 128]	17.78 hours
3	Normalisation: zscore	Kidney 2 (1041,1511,2217) & Kidney 3 (1706x1510,501)	Kidney 1 (1303,912,2279)	[1510.5, 1373.5, 1359.0]	2	[128, 128, 128]	17.78 hours
4	Normalisation: zscore	Half of Kidney 1 (1303,912,1139)	The other half of kidney 1 (1303,912,1140)	[1303.0,912.0, 1139.0]	2	[1303,912, 1139]	17.01 hours

Supplementary Figures

Supplementary Figure 1: Proposed automated method configuration for nnU-Net based biomedical image segmentation –from original nnU-Net paper [2].

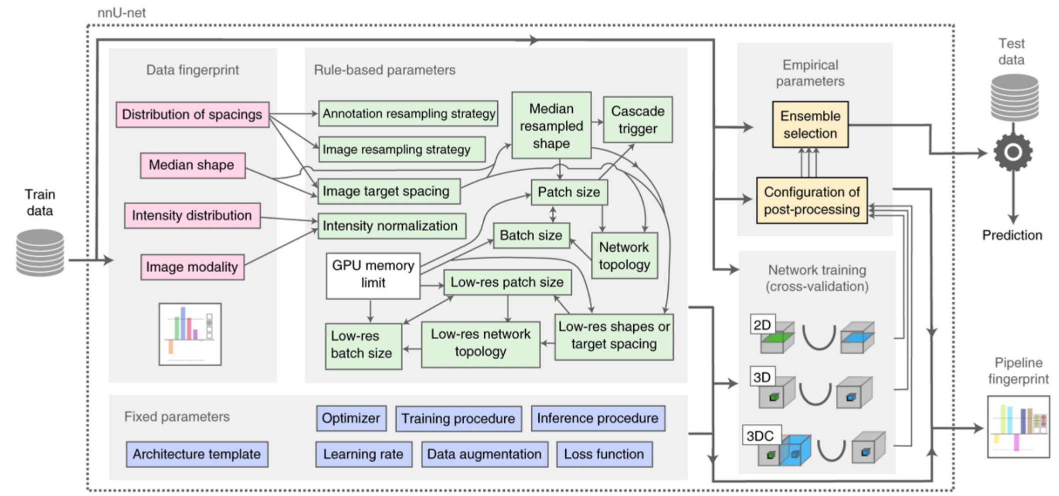

Design choice	Required input	Automated (fixed, rule-based or empirical) configuration derived by distilling expert knowledge (more details in online methods)
Learning rate	–	Poly learning rate schedule (initial, 0.01)
Loss function	–	Dice and cross-entropy
Architecture template	–	Encoder–decoder with skip-connection ('U-Net-like') and instance normalization, leaky ReLU, deep super-vision (topology-adapted in inferred parameters)
Optimizer	–	SGD with Nesterov momentum (μ = 0.99)
Data augmentation	–	Rotations, scaling, Gaussian noise, Gaussian blur, brightness, contrast, simulation of low resolution, gamma correction and mirroring
Training procedure	–	1,000 epochs × 250 minibatches, foreground oversampling
Inference procedure	–	Sliding window with half-patch size overlap, Gaussian patch center weighting
Intensity normalization	Modality, intensity distribution	If CT, global dataset percentile clipping & z score with global foreground mean and s.d. Otherwise, z score with per image mean and s.d.
Image resampling strategy	Distribution of spacings	If anisotropic, in-plane with third-order spline, out-of-plane with nearest neighbor Otherwise, third-order spline
Annotation resampling strategy	Distribution of spacings	Convert to one-hot encoding → If anisotropic, in-plane with linear interpolation, out-of-plane with nearest neighbor Otherwise, linear interpolation

Image target spacing	Distribution of spacings	If anisotropic, lowest resolution axis tenth percentile, other axes median. Otherwise, median spacing for each axis. (computed based on spacings found in training cases)
Network topology, patch size, batch size	Median resampled shape, target spacing, GPU memory limit	Initialize the patch size to median image shape and iteratively reduce it while adapting the network topology accordingly until the network can be trained with a batch size of at least 2 given GPU memory constraints. for details see online methods.
Trigger of 3D U-Net cascade	Median resampled image size, patch size	Yes, if patch size of the 3D full resolution U-Net covers less than 12.5% of the median resampled image shape
Configuration of low-resolution 3D U-Net	Low-res target spacing or image shapes, GPU memory limit	Iteratively increase target spacing while reconfiguring patch size, network topology and batch size (as described above) until the configured patch size covers 25% of the median image shape. For details, see online methods.
Configuration of post-processing	Full set of training data and annotations	Treating all foreground classes as one; does all-but-largest-component-suppression increase cross-validation performance? Yes, apply; reiterate for individual classes No, do not apply; reiterate for individual foreground classes
Ensemble selection	Full set of training data and annotations	From 2D U-Net, 3D U-Net or 3D cascade, choose the best model (or combination of two) according to cross-validation performance

Supplementary Figure 2: Training progress for experiment 1.

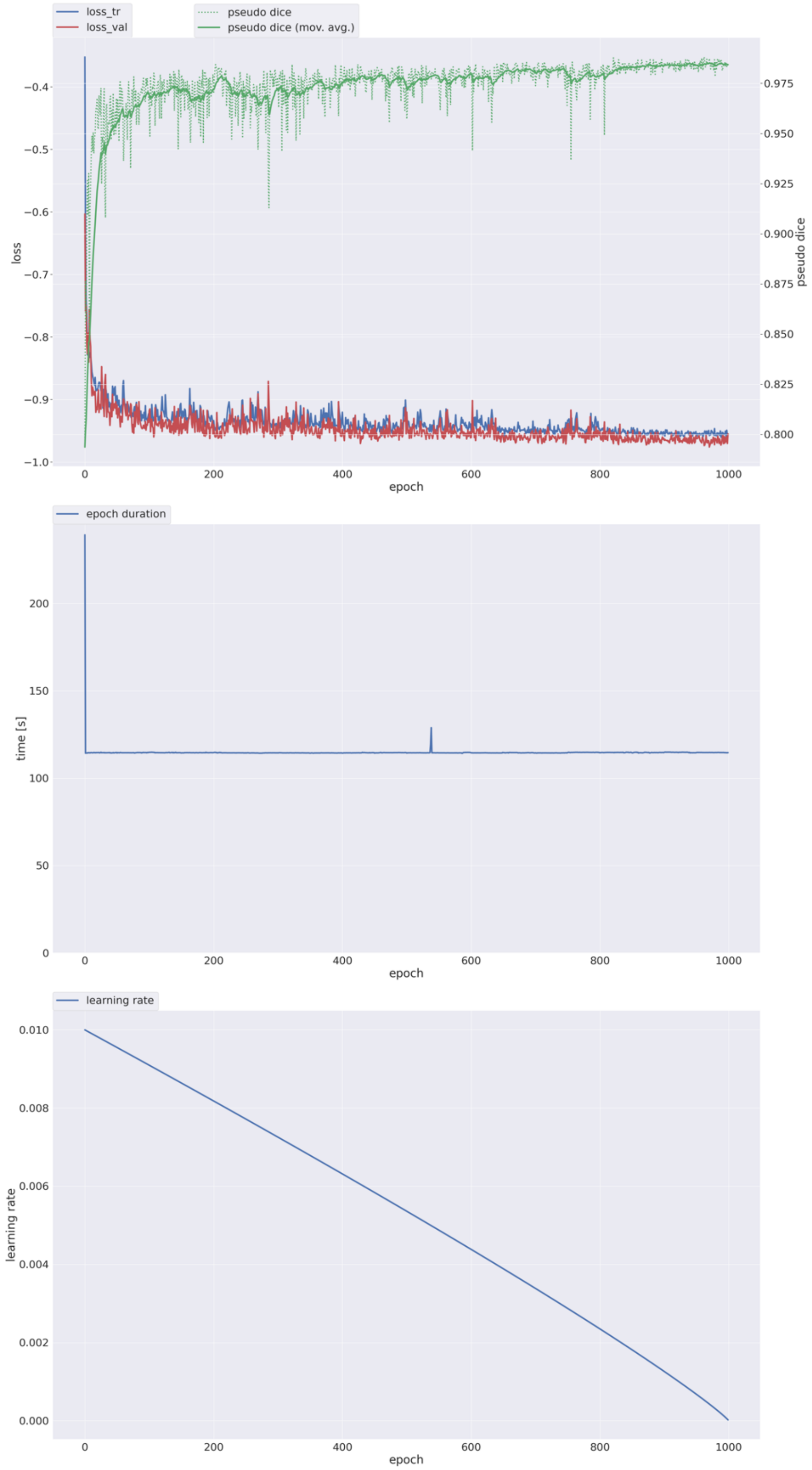

Supplementary Figure 3: Training progress for experiment 2.

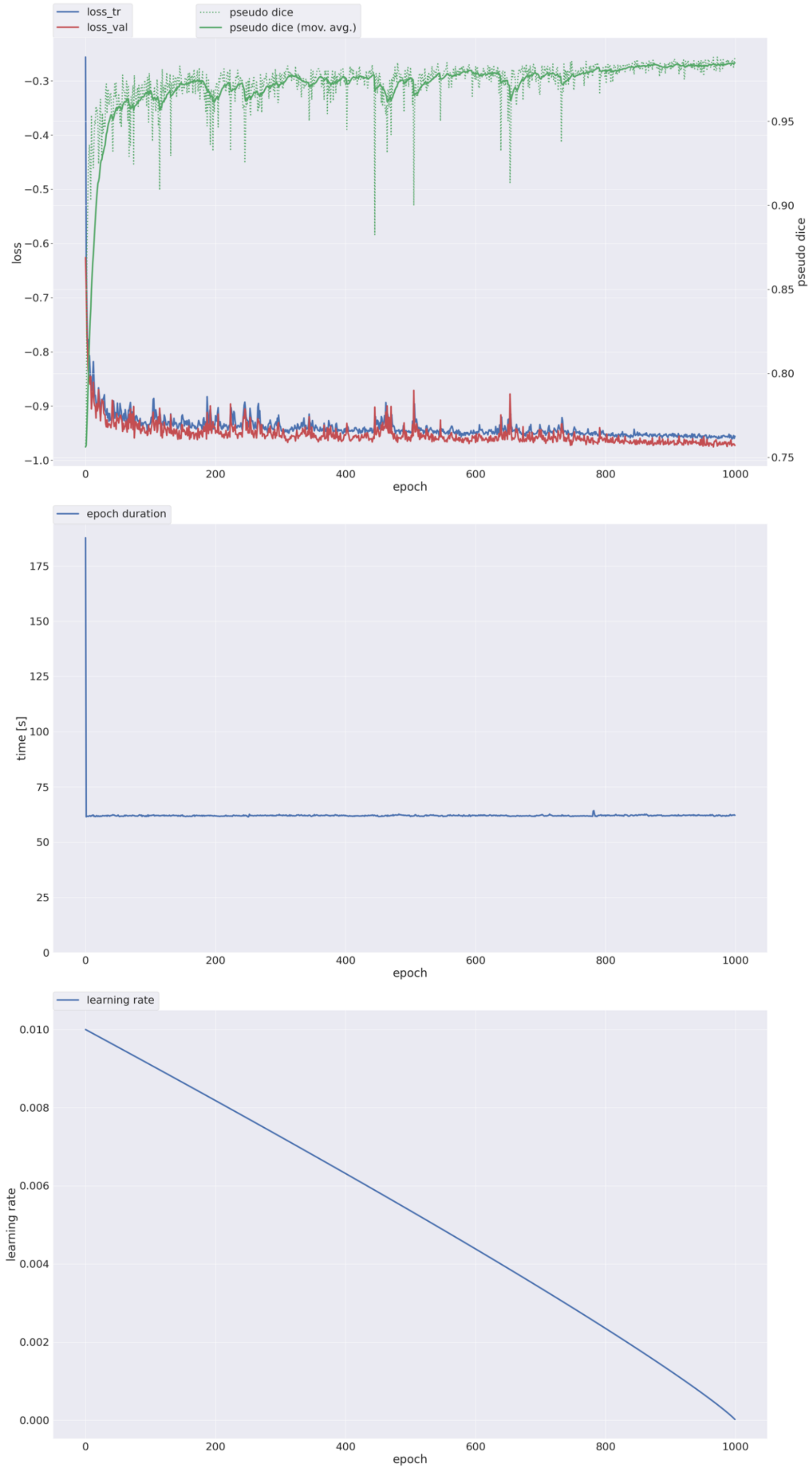

Supplementary Figure 4: Training progress for experiment 3.

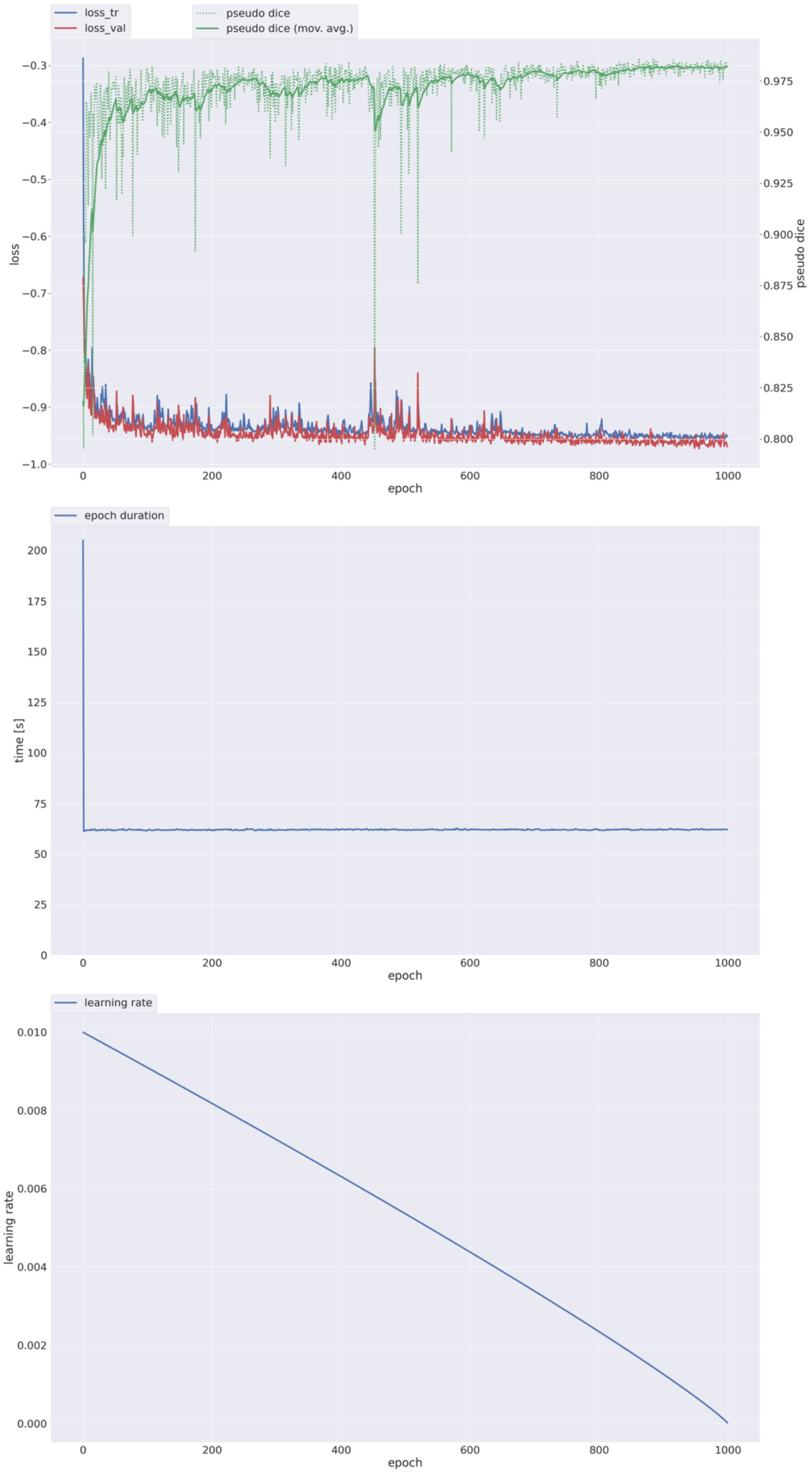

Supplementary Figure 5: Showing the skeleton that is produced during the cl-Dice computation in Experiment 3, the inset shows the ball like structures that can occur and disrupt the metric output.

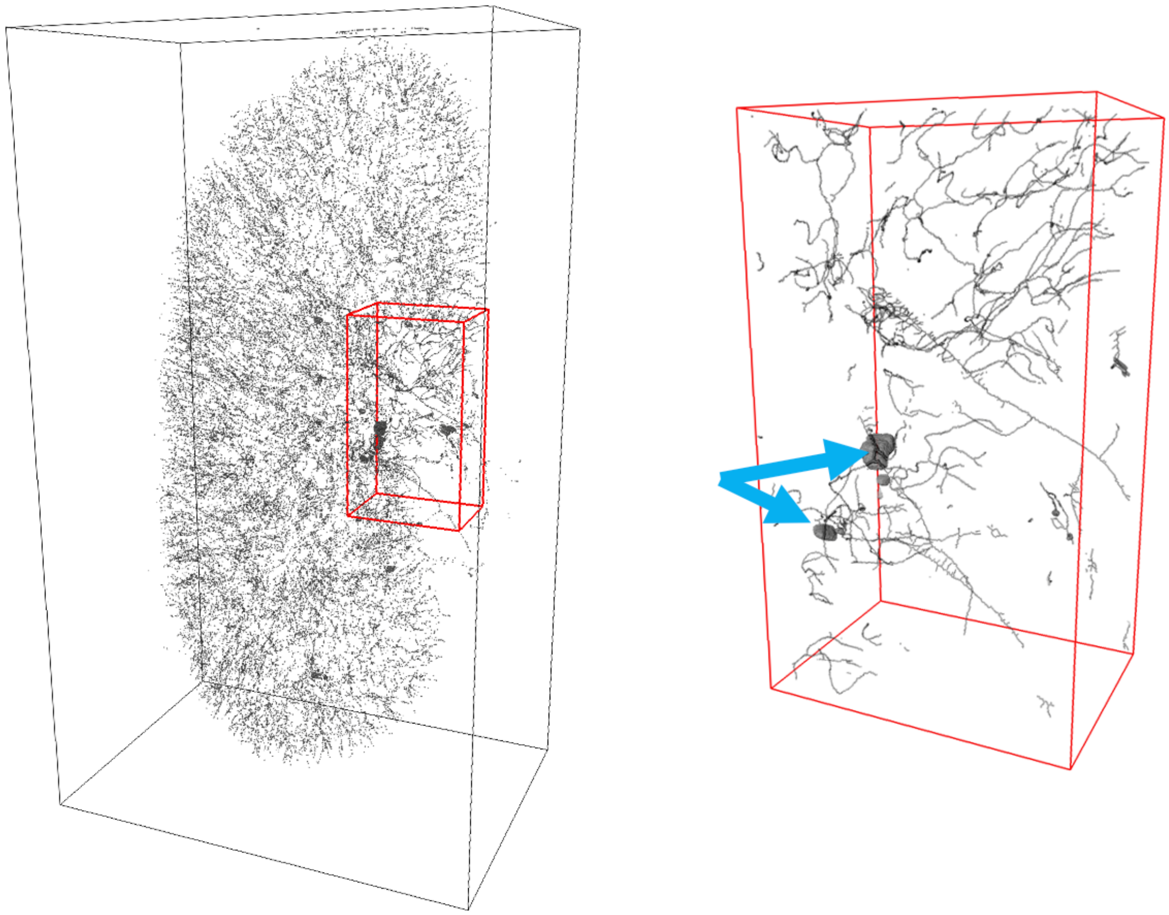

Supplementary Figure 6: Showing some examples where false positives align with actual anatomical structures in the corresponding 2D ortho slice.

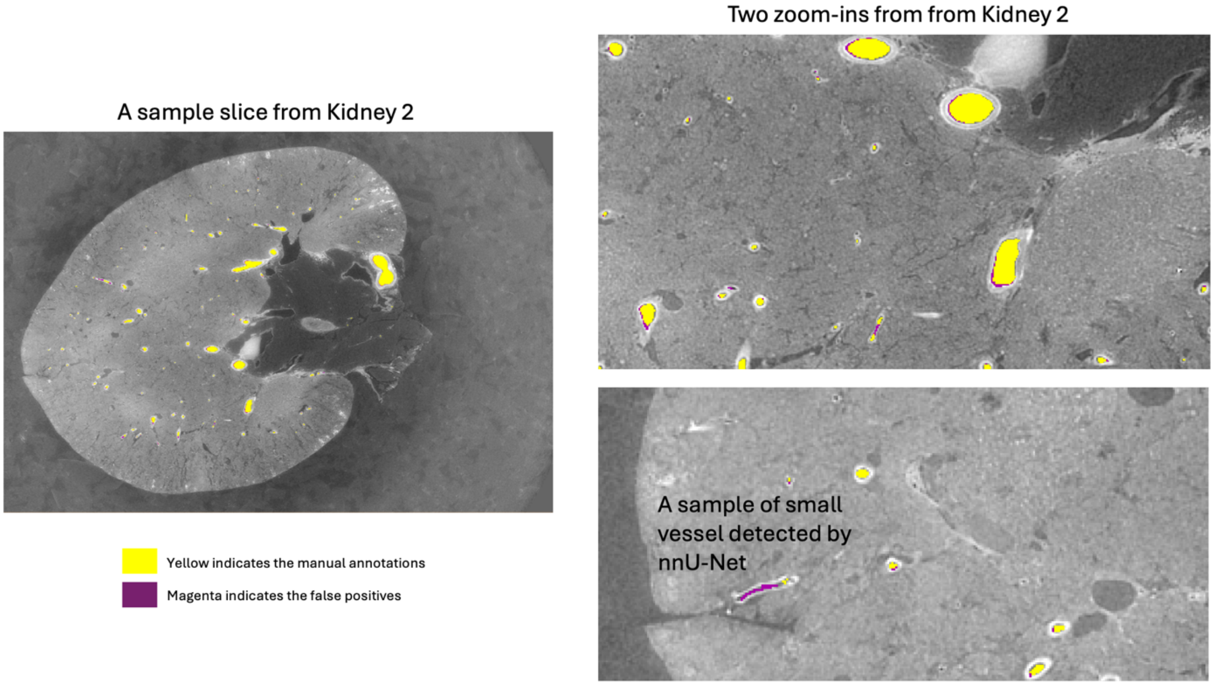

References

[1] Wu, Mian, et al. "Hepatic vessel segmentation based on 3D swin-transformer with inductive biased multi-head self-attention." *BMC Medical Imaging* 23.1 (2023): 91.

[2] Isensee, Fabian, et al. "nnU-Net: a self-configuring method for deep learning-based biomedical image segmentation." Nature methods 18.2 (2021): 203-211.
